# Galectin-1-Binding Glycoforms of Haptoglobin with Altered Intracellular Trafficking, and Increase in Metastatic Breast Cancer Patients

**DOI:** 10.1371/journal.pone.0026560

**Published:** 2011-10-18

**Authors:** Michael C. Carlsson, Cecilia Cederfur, Viveka Schaar, Crina I. A. Balog, Adriana Lepur, Franck Touret, Emma Salomonsson, André M. Deelder, Mårten Fernö, Håkan Olsson, Manfred Wuhrer, Hakon Leffler

**Affiliations:** 1 Section MIG (Microbiology, Immunology, Glycobiology), Department of Laboratory Medicine, Lund University, Lund, Sweden; 2 Biomolecular Mass Spectrometry Unit, Department of Parasitology, Leiden University Medical Center, Leiden, The Netherlands; 3 Department of Oncology, Lund University Hospital, Lund, Sweden; University of Central Florida, United States of America

## Abstract

Sera from 25 metastatic breast cancer patients and 25 healthy controls were subjected to affinity chromatography using immobilized galectin-1. Serum from the healthy subjects contained on average 1.2 mg per ml (range 0.7–2.2) galectin-1 binding glycoproteins, whereas serum from the breast cancer patients contained on average 2.2 mg/ml (range 0.8–3.9), with a higher average for large primary tumours. The major bound glycoproteins were α-2-macroglobulin, IgM and haptoglobin. Both the IgM and haptoglobin concentrations were similar in cancer compared to control sera, but the percentage bound to galectin-1 was lower for IgM and higher for haptoglobin: about 50% (range 20–80) in cancer sera and about 30% (range 25–50) in healthy sera. Galectin-1 binding and non-binding fractions were separated by affinity chromatography from pooled haptoglobin from healthy sera. The N-glycans of each fraction were analyzed by mass spectrometry, and the structural differences and galectin-1 mutants were used to identify possible galectin-1 binding sites. Galectin-1 binding and non-binding fractions were also analyzed regarding their haptoglobin function. Both were similar in forming complex with haemoglobin and mediate its uptake into alternatively activated macrophages. However, after uptake there was a dramatic difference in intracellular targeting, with the galectin-1 non-binding fraction going to a LAMP-2 positive compartment (lysosomes), while the galectin-1 binding fraction went to larger galectin-1 positive granules. In conclusion, galectin-1 detects a new type of functional biomarker for cancer: a specific type of glycoform of haptoglobin, and possibly other serum glycoproteins, with a different function after uptake into tissue cells.

## Introduction

**Figure 1:**
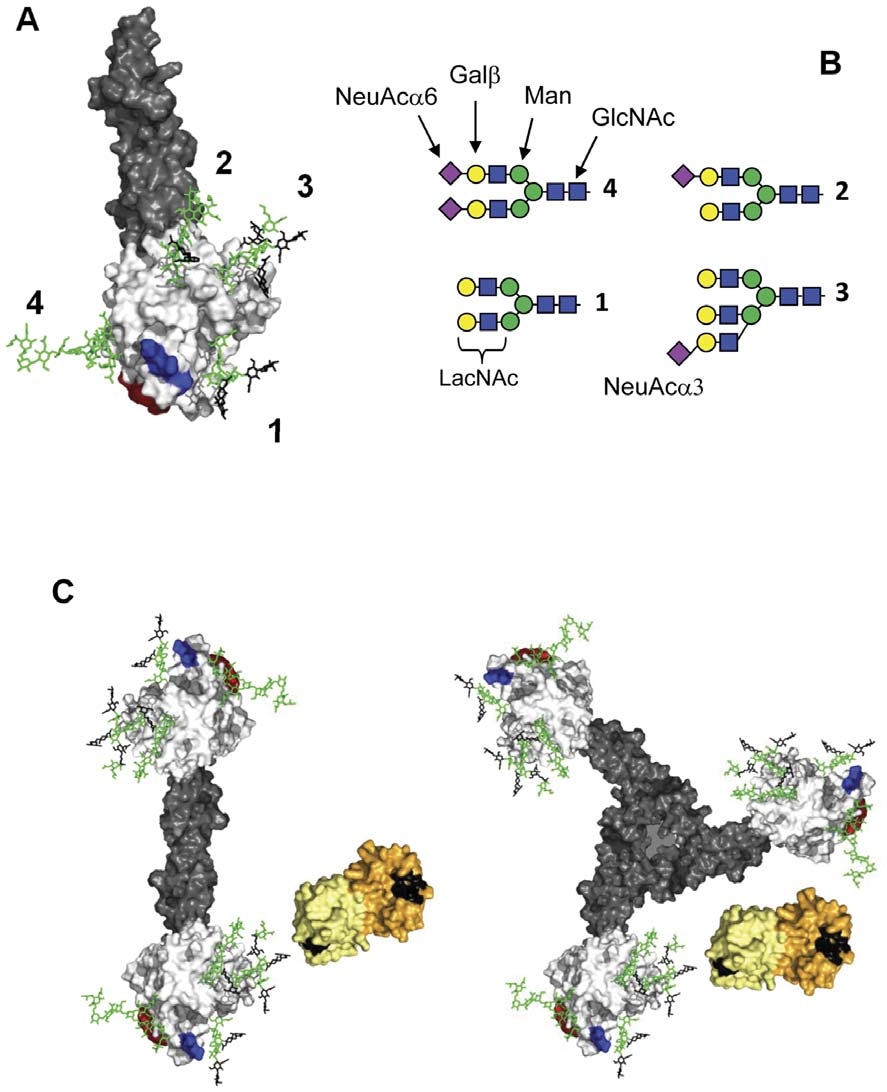
Haptoglobin and galectin-1. **A**) A model of one glycoform of haptoglobin (Hp2) with the α-subunit in grey and β-subunit in white, and N-glycans attached at the four known sites (numbers) [2], [3] shown as stick models in green but with the LacNAc parts potentially binding galectin-1 in black. A surface thought to interact with haemoglobin is shown in red and the CD163-binding site is shown in blue [65]. **B**) Schematics of the N-glycans shown in panel A, with component saccharide moieties indicated. The major N-glycan of human serum is no. 4, a biantennary N-glycan capped with NeuAcα6 (6 bound sialic acid, shown as purple diamonds), which would block galectin binding [20]. The other glycans may lack one or more of the NeuAcα6, and/or have an additional antenna with a NeuAcα3, all of which would open potential galectin-binding sites (see Table S3 in Cederfur et al. [25]). **C**) Models of dimeric haptoglobin Hp1 and trimeric haptoglobin Hp2, built from the model of panel A, together with two human galectin-1 dimers (pdb-id: 1W6P), colored with the two subunits in different shades of yellow, and the LacNAc-binding site (C–D, [20]) in black. The monosaccharide symbols are as recommended by Consortium for Functional Glycomics (http://glycomics.scripps.edu/CFGnomenclature.pdf): purple diamond, N-acetylneuraminic acid; blue square, N-acetylglucosamine; green ball, mannose; yellow ball, galactose. The coordinates for the model of the haptoglobin peptide chains (panel A) were obtained from the SWISS-MODEL REPOSITORY(http://swissmodel.expasy.org/repository/), Model_id: 76a3bb6963a6b1ee5c09942c719f20c2_UP000020_3, which in turn is built based on the X-ray crystal structure of complement protein C1r (pdb-id: 1GPZ). Four N-glycans with structures as shown in Fig. 1C were attached to the four known N-glycosylation sites of haptoglobin and built into this model and energy minimized using the server GlycamWeb (http://glycam.ccrc.uga.edu/ at Complex Carbohydrate Research Center, The University of Georgia, Athens, GA). The models were visualized using The PyMOL Molecular Graphics System, Version 1.3, Schrödinger, LLC. For the haptoglobin dimer and trimer of panel C, two or three copies of the model of panel A were manually arranged to resemble the models published by Polticelli et al. [1] regarding distances between the β-subunits.

A glycoprotein occurs in multiple glycoforms depending on which glycans are attached at each particular site. Haptoglobin, for example (Fig. 1) [1], consist of two chain subunits (αβ) with four N-glycosylation sites (Fig. 1A) [2], [3], which in turn can form dimers, trimers or higher oligomers giving each complete molecule eight, twelve or more N-glycosylation sites (Fig. 1C). Each of these sites can carry one out of a big collection of different N-glycans (Fig. 1B shows four examples of the over 100 known in human serum), making the total number of different possible glycoforms very large. The composition and proportion of all these different glycoforms are not random, however, but are strikingly constant over time in each healthy individual [4], and also vary little between most individuals in a population [5], suggesting tight physiological regulation and function.

The glycan structures, and thereby the profile of glycoforms of different glycoproteins, have been known for a long time to be altered in cancer [6], [7]. This has stimulated an increasing effort to use particular glycoforms as biomarkers for cancer in serum, as detected by combinations of plant lectins, antibodies and structural analysis by mass spectrometry, summarized as glycoproteomics [8], [9]. These may be derived from the cancer itself [8], [10], [11], [12], and in fact some of the most commonly used cancer biomarkers are carbohydrate based and detection of specific glycoforms of other commonly used cancer associated proteins, such as PSA, have been proposed to sharpen the diagnosis. Specific cancer induced forms of common serum glycoproteins, such as transferrin or haptoglobin that are synthesized mainly in the liver, have also been observed and may serve as markers of the physiological effects of the cancer [2], [3], [13], [14], [15].

The functional effects of the cancer-related carbohydrate changes, however, have been more elusive. One hypothesis has been that cancer associated carbohydrate structures modulate cell adhesion, e.g. sialyl-Lewis X-containing glycans bind to endothelial carbohydrate binding proteins, selectins, to promote metastasis [8], [16]. Another recent hypothesis is that cancer associated carbohydrate structures modulate intracellular traffic of a glycoprotein via interaction with another family of carbohydrate binding proteins, the galectins. For example, tri- and tetraantennary N-glycans bind galectin-3 to increase cell surface residence time of epidermal growth factor receptors in cancer cells, in turn increasing cell sensitivity and growth response to EGF [6], [17], and by analogous mechanisms, galectin-1 regulates cell surface expression of integrins [18], in turn affecting tumour cell adhesion and migration, and cell surface expression of the calcium channel TRPV5, in turn affecting Ca-homeostasis [19].

Galectins are a family of small animal proteins binding specific carbohydrate chains containing β-galactosides, such as N-acetyllactosamine (LacNAc) (Fig. 1B and C) [20], [21]. Largely independent of the research on cancer carbohydrates described above, a number of possible relationships between galectins and cancer, inflammation and immunity have been suggested, with proposed effects on cell adhesion, angiogenesis, apoptosis and various types of signaling [22], [23], [24]. Currently, the role in intracellular trafficking of glycoproteins, described above, provides a link between galectins and carbohydrate structures related to cancer and also other pathophysiological conditions [6], [17].

Here we propose that this link between galectins and particular glycans may also apply to serum glycoproteins, and provide the basis for a new type of functional biomarkers. Almost all potential galectin binding sites on serum glycoproteins have a LacNAc residue as a central part (Fig. 1B). However, LacNAc by itself binds galectin with relatively low affinity (K_d_∼100 µM). Moreover, most of the LacNAc residues on serum glycoproteins are capped by 2–6 sialic acid that prevents binding of all galectins as in the major serum N-glycan (upper left structure in Fig. 1B). The remainders of the LacNAc residues bind different galectins to a different extent depending on the details of their structural context, i.e. neighboring saccharides and protein parts that can enhance or decrease binding affinity, and each galectin can be used as a reagent to detect different glycoforms. Galectin-3 binds relatively large fractions of a wide range of serum glycoproteins, whereas galectin-1 binds a smaller fraction of a more restricted set and galectin-2 binds none at all [25].

Now we show that in sera from patients with metastatic breast cancer, galectin-1 binds on average almost twice as much glycoprotein compared with healthy individuals, including a fraction of haptoglobin containing N-linked glycans with less terminal sialic acids, and increased proportion of additional antenna (as in the bottom right structure of Fig. 1B). Moreover, the galectin-1 bound glycoforms of haptoglobin had different trafficking, compared to the non-bound haptoglobin, after uptake into macrophages (in complex with haemoglobin). Thus galectin-1 detects a specific subset of this serum protein, which occurs at an increased level in sera from cancer patients and has a different function in tissue cells.

## Materials and Methods

### Patient samples

Deidentified human serum samples were used, under ethical permit to authors HO and MF and approval from the Ethical Review Board at Lund University (Now Regional Ethical Review Board Lund, http://www.epn.se/lund/om-naemnden.aspx). Written informed consent was obtained from all participants. All information and data was handled confidentially, and evaluation of information linked to patients was carried out in accordance with the Swedish Personal Data Act (Personuppgiftslagen in Swedish). Serum samples from 25 female metastatic breast cancer patients and 25 age matched healthy female volunteers were collected and stored as described previously [25], [26]. Subject age and pathology diagnosis are shown in Table S1.

### Production of recombinant galectin

The cDNA encoding the C3S mutant of human galectin-1 was generously provided by Dr. Jun Hirabayashi [27]. Different mutants were made from this cDNA and confirmed by sequencing by the same procedures as reported previously [28]. The recombinant proteins were produced in *E. coli* BL21 Star and purified by chromatography on lactosyl-Sepharose as previously described [25].

### Galectin affinity chromatography

Galectins were coupled to 1 ml NHS-activated Hi-Trap affinity columns (Amersham Biosciences) as described by Cederfur *et al.* [25]. While kept on ice, 0.1 ml serum in 1.9 ml PBS or 2 mg of haptoglobin (pooled from human plasma, Sigma-Aldrich) in 2 ml PBS was circulated for 30 minutes at 1 ml/min on the 1 ml galectin-1 C3S column. The column was sealed for 30 minutes to enhance the ligand-galectin binding. The unbound fraction (flow-through) was saved and the column was washed with 32 column volumes of PBS. Galectin binding proteins were eluted with 150 mM lactose in PBS, in fractions of 0.2 ml, up to five column volumes. Protein concentrations were determined with the Bio-Rad protein assay and fractions were kept and stored at −20°C until further analysis. Six sera from both cancer patients and healthy controls were analyzed a second time to evaluate the method constancy, resulting in a within sample variation of <8%. A duplicate of a standard serum sample was rechromatographed about each 10th run, to ensure that column performance remained within this range; thus each column was used for approximately 15 chromatographed sera. Some columns were opened after the last use, and an aliquot from the top of the column gel was removed, boiled in sample buffer and analyzed by SDS-PAGE (as described for galectin-3 columns in [25]), to show that no protein was retained after lactose elution. There was also no evidence that the column binding capacity was exceeded, since when selected flow through fractions were rechromatographed, no additional glycoproteins bound. Moreover, the column could isolate 0.6 mg haptoglobin without sign of overload as described under results, whereas the amount bound from serum samples was between 0.07 and 0.4 mg.

### Fluorescence anisotropy assay

Fluorescence anisotropy assay was used to compare the direct binding of different fluorescein tagged saccharide probes to galectin-1 and mutants, and to compare the potency of small saccharides and glycoproteins to inhibit the interaction of galectin-1 and mutants with a high affinity thiodigalactoside-amid (tdga)-probe, as described in detail [28].

### SDS-PAGE, western blotting and proteomics

Serum ligands were analyzed by one-dimensional 4–20% SDS-PAGE, western blotting, and MALDI-TOF of tryptic digests of excised bands as described previously [25].

### Lycopersicon Esculentum affinity chromatography

0.1 ml of human serum were added to a 1 ml column of agarose bound Lycopersicon Esculentum (Vectorlabs) followed by extensive wash (30 column volumes) with PBS. 0.3 ml flow through fractions were collected and presence of protein was determined using Bio-Rad protein assay. Protein containing samples were pooled and run on a galectin-1 C3S column as described earlier. 0.1 ml of column material was removed and boiled for 5 minutes before analyzed with SDS-PAGE as described above.

### Neuraminidase treatment of serum and transferrin

0.1 ml serum or 2 mg of human transferrin (Sigma- Aldrich) was treated with neuraminidase from *Vibrio cholerae* (Roche) (25 µmol per µmol of transferrin) for 1 h at 37°C. Treated and untreated samples were then separated by affinity chromatography as described above.

### Haptoglobin ELISA

Haptoglobin was quantitated in microtiter well immobilized samples using biotinylated goat α-haptoglobin (Sigma Aldrich, EZ-Link® Sulfo-NHS-SS Biotinylation Kit, Pierce) followed by Streptavidin horseradish peroxidase (HRP) conjugate for detection. Three independent repeat ELISA experiments, each in duplicate, were performed in 96 well PolySorb microtitre plates (Nunc). All wells except two (blank), were incubated with 100 µl of either unseparated serum samples (1∶90), pooled galectin-1 bound fractions (1∶18) or a dilution series of haptoglobin (pooled from human plasma, Sigma-Aldrich) (Starting at 0.2 with 1∶2 dilutions) at 4°C for 24 hours. After coating, wells were blocked with 100 µl 1% BSA for 1 hour at 37°C and then 100 µl of biotin conjugated goat α-human haptoglobin (1∶1000) were added to each well. After 1 hour incubation at RT, wells were washed 4 times with 0.05% tween in PBS and incubated for 1 hour with 50 µl of Streptavidin horseradish peroxidase (HRP) conjugate (1∶5000) at RT. The wells were washed 4 times with 0.05% tween in PBS and presence of HRP was detected with TMB Peroxidase EIA substrate kit (BioRad). Absorbance at 650 nm was measured by using Labsystems multiscan RC plate reader (Labsystems). Blank value was subtracted from each sample and haptoglobin content of each serum was calculated from standard curve using nonlinear regression in Prism 5.03 (GraphPad Software).

### IgM-ELISA

IgM was quantitated in immobilized samples (two independent repeat experiments with duplicate samples in each) by the same procedure as described for haptoglobin above, except that detection was with peroxidase conjugated α-human IgM (1∶1000) (Sigma-Aldrich), and a dilution series of serum with known IgM concentration was used to construct the standard curve.

### Galectin-1competitive ELISA

Galectin-1 was quantitated in serum from 3 cancer patients (# 1, 2 and 4) and 2 healthy controls (# 1 and 2) in 96 well plates coated with 2 ng/µl α-rat-galectin-1 (BAbCO) antibody. Biotin labeled recombinant galectin-1 was used as a competitor in a concentration of 50 ng/ml. For galectin-1 biotinylation, EZ-link sulfo-NHS-SS- Biotinylation kit (Pierce) was used as instructed. 1–500 ng/ml of recombinant galectin-1 was used to make an inverse standard curve. Bound biotin-galectin-1 was recognized by 1∶10000 diluted avidin-HRP (BioRad). Presence of HRP was detected with TMB Peroxidase EIA substrate kit (BioRad) Absorbance at 650 nm was measured by using Labsystems multiscan RC plate reader (Labsystems).

### Statistical analysis and ROC-plots

All pair wise comparisons between the cancer and control sera were done by a two tailed unpaired t-test, and receiver operating characteristic (ROC) curves were constructed using the Prism software.

### Glycan analysis

The bound and unbound haptoglobin fractions (approximately 30 µg per line) were separated by SDS-PAGE under reducing conditions on Novex 4–12% Bis-Tris gradient gels (Invitrogen). Total protein was visualized by the colloidal blue staining kit according to the instructions of the manufacturer (Invitrogen). The excised β-haptoglobin bands were incubated overnight at 37°C with 8 µl of PNGase F (0.5 mU) in 30 µl of 25 mM ammonium bicarbonate to allow *N*-glycan release. N-glycans were separated using an Ultimate 3000 nano-LC system (Dionex/LC Packings) equipped with a graphitized carbon trap column (Hypercarb, 5 µm, 170 µm×10 mm; Thermo Scientific) and a nano column (75 µm×100 mm) packed by Grom Analytik. The column was equilibrated at room temperature with eluent A (0.1% formic acid in water) at a flow rate of 400 nL min^−1^. After injection of the sample a linear gradient was applied (15 min 25% eluent B (95% acetonitrile), 25 min 70% B, 30 min 70% B). The eluate was monitored by absorption at 215 nm. The LC column was coupled to an Esquire HCT-Ultra ESI-ion trap-MS (Bruker-Daltonics, Bremen, Germany) equipped with an online nanospray source operated in the positive-ion mode. For electrospray (1100–1250 V), electropolished, stainless steel LC−MS emitters (150 µm OD, 30 µm ID) from Proxeon A/S were used. The solvent was evaporated at 170°C employing a nitrogen stream of 6 L min^−1^. Ions from *m*/*z* 500 to *m*/*z* 1800 were monitored in the MS mode. When operated in the auto MS/MS mode, monitoring ions from *m*/*z* 140 to 2200, each MS scan was followed by the acquisition of MS/MS spectra of up to five of the most abundant ions in the MS spectrum. Glycan structures were assigned using GlycoWorkbench [29].

### Glycopeptide analysis

Alternatively to the glycan analysis procedure, SDS-PAGE gel pieces containing β-haptoglobin were reduced, alkylated and digested with trypsin (Promega), as previously described [30]. After digestion, (glyco-) peptides were collected using two rounds of extraction with 20 µl of 0.1% TFA and stored at −20°C prior to analysis by mass spectrometry. Total tryptic digests of bound and unbound β-haptoglobin were applied to a reverse-phase column (PepMap, 3 µm, 75 µm·100 mm; Dionex/LC Packings) using an Ultimate 3000 nano-LC system (Dionex/LC Packings). The column was equilibrated at room temperature with eluent A (0.1% formic acid in water) at a flow rate of 300 nL min^−1^. After injection of the sample a linear gradient was applied (15 min 25% eluent B (95% acetonitrile), 25 min 70% B, 30 min 70% B). The eluate was monitored by absorption at 215 nm. Eluates were analyzed by ESI-ion trap-MS as described above.

### Haptoglobin-haemoglobin complex

Haptoglobin (galectin-1 binding or non-binding) and haemoglobin were added in a 1∶1 weight ratio (0. 28 µg of each or 0.23 µmoles of haptoglobin and 0.40 µmoles of haemoglobin) and left shaking for 10 minutes at room temperature in order to allow formation of complexes. Formation of haptoglobin-haemoglobin complexes were analyzed using a native gel electrophoresis (without β-mercaptoethanol and SDS). Samples of approximately 10 µg of protein were added to a 4–20% polyacrylamide gel (Pierce).

### Labeling of proteins

A 15 fold access of labeling dye; NHS-Fluorescein or NHS-Sulphorhodamine (Thermo Scientific) dissolved in DMF was added to a 2 mg/ml protein solution in a 100 mM carbonate/bicarbonate buffer. The solution was mixed well and incubated for 1 h at room temperature. Labeled protein was separated from the unreacted dye by a buffer change to PBS on a PD10 column (Amersham Biosciences).

### Cell lines and cell cultures

THP-1 (Human acute monocytic leukemia cell line) (ATCC) cells were kept in incubators at 37°C and 5% CO2. The cells were grown in 24-well plates with RPMI-1640, supplemented with 10% Foetal Calf Serum and 1% Penicillin-Streptomycin (Invitrogen). In order to stimulate monocytes to differentiate into macrophages, THP-1 cells were grown in media additionally containing 100 ng/ml phorbol-12-myristate-13-acetate (PMA) (Sigma-Aldrich) for five days and 10 ng/ml Il-4 for 2 days. Untreated cells were transferred to a polylysine glass slide and allowed to sediment for 15 min in room temperature, while treated cells were grown directly on coverslips.

### Haptoglobin endocytosis in differentiated THP-1 cells

THP-1 cells were grown in 24-well plates containing coverslips and were treated as described above. Cells were incubated for 30 min in serum-free medium followed by incubation with 0.2 µM rhodamine conjugated haptoglobin-haemoglobin complex in serum free medium at 37°C. Control experiments were performed using 0.2 µM of rhodamine conjugated haptoglobin or haemoglobin. Endocytosis was stopped at different time points by removing the medium followed by addition of ice cold PBS and fixation in 2% formaldehyde. Cells were then permeabilized with 500 µl 1% Triton-X in PBS for 10 min and washed with PBS. Unspecific binding was prevented using blocking buffer (0.1% Tween, 1% FBS in PBS) for 10 minutes at room temperature. Immunostaining was performed using either of mouse α-human-LAMP-2(α-CD107a, PharMinogen), rabbit α-rat-galectin-1 (BAbCO), mouse α-human CD163 (eBioscience) or goat α-human-haptoglobin (Sigma-Aldrich) at a ratio of 1∶100 for 1 h at room temperature in a moist chamber. Cells were washed with PBS and then incubated with corresponding secondary antibody α-mouse Alexa Fluor488, α-rabbit Alexa Fluor488 and α-goat Alexa594 (Molecular Probes, Invitrogen) at a ratio of 1∶320. Control staining with only secondary antibody was performed for each experiment. Coverslips were mounted using Moviol with 0.01 mg/ml Hoechst 33342 (Fluka).

### Image Acquisition

Phase contrast or epifluorescent images were captured using a Nikon eclipse TE2000-U fluorescence microscope with a Plan Apochromat ×40 or a CFI Plan Apochromat ×100 oil objective: numeric aperture 1.40, working distance 0.13 mm, and equipped with a digital still camera DS-Qi1MC. Fluorescent images (RGB, 8 bit, 1280×1024) were deconvolved from a 3D stack (x,y,z dimensions: 129.82×103.85×21.6 µm) with NIS-element AR software. 5 images of each individual experiment were examined using NIS-element AR software colocalization application and average values of overlap coefficients according to Manders were calculated after background correction.

## Results

### Affinity chromatography of serum samples on immobilized galectin-1

**Figure 2:**
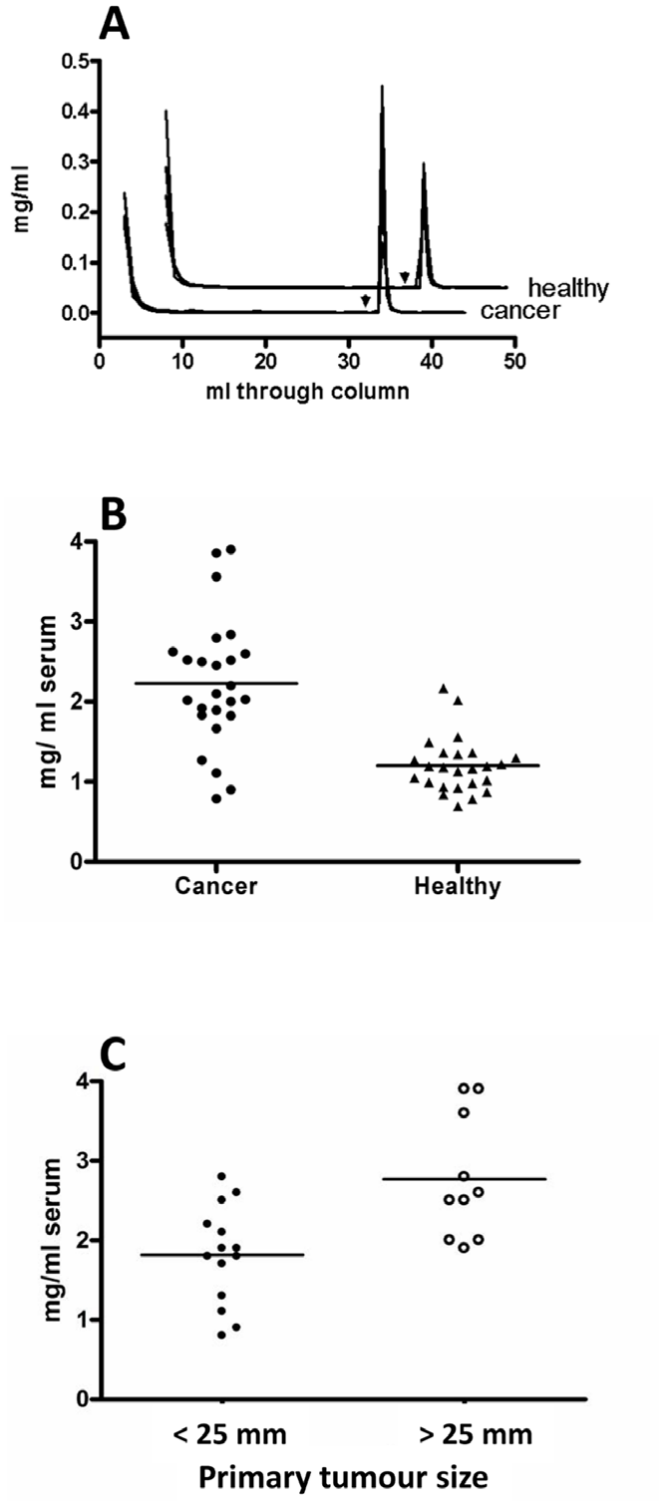
Affinity chromatography of cancer and healthy sera on immobilized human galectin-1 C3S. **A**) Superimposed chromatograms from 5 cancer sera and 4 healthy sera. The protein concentration of each fraction (0.2 or 1 ml) is given on Y-axis. Elution of bound fractions with lactose (150 mM) started after washing with 32 ml PBS (arrow heads). The chromatograms from healthy sera have been moved for clarity by +0.05 on Y-axis and by +5 on X-axis. **B**) Yield of galectin-1-binding proteins (sum of protein amount in bound fractions multiplied by 10 to give mg per ml original serum) for sera from 25 cancer patients and 25 healthy. The average for each group is marked by horizontal lines, and the difference was statistically significant (p<0.0001). **C**) Yield of galectin-1-binding proteins from 24 cancer patients divided based on primary tumour size. The average for each group is marked by horizontal lines, and the difference was statistically significant (p = 0.0028).

Multiple human serum samples (0.1 ml) were subjected to affinity chromatography on columns (1 ml) with immobilized human galectin-1. To improve stability, a C3S mutant of human galectin-1 was used, which is less sensitive to inactivation by oxidation than wild type galectin-1 [31], and previously shown to have the same affinity for carbohydrates and glycoproteins, including human serum glycoproteins, as wild type galectin-1 [28]. Typical chromatograms are shown in Fig. 2A, demonstrating that unbound proteins pass through the column without trailing (since the curve goes down to zero during washing), and a well-defined fraction of bound serum glycoproteins withstands the extensive washing with buffer (32 column volumes) and can be eluted as a sharp peak with buffer containing the competitive ligand lactose. Modeling of the column performance [25] shows that a binding ligand with this behavior needs an affinity of K_d_∼1–5 µM or better for galectin-1. In addition, the amount of serum ligand with this affinity for galectin-1 was confirmed by a fluorescence anisotropy assay as described in detail for galectin-1 [28] and galectin-3 [25].

### Galectin-1 binds increased levels of glycoproteins in sera from metastatic breast cancer patients

Twenty-five sera from women with metastatic breast cancer (#1–25 in supportive material Table 1, median age 64 years) and 25 sera from healthy women (#26–50, median age 65 years) were analyzed by affinity chromatography on immobilized galectin-1 as exemplified for some in Fig. 2A. The yield of galectin-1 binding proteins from the healthy sera was on average 1.2 mg per ml serum (range 0.7–2.2), whereas the yield from the cancer sera was significantly (p<0.0001) higher with an average of 2.2 mg per ml serum (range 0.8–3.9) (Fig. 2B). The yields from the cancer patients were compared to various aspects of the available clinical data for each patient (Supportive material Table 1) including pharmacological treatments (not shown). The only correlation found was with tumour size (Fig. 2C); sera from patients with larger primary tumours (>25 mm) gave significantly higher average yields of galectin-1 bound glycoproteins (2.8 mg/ml serum, p = 0.0028) than smaller primary tumours (<25 mm, 1.8 mg per ml serum). Sera from patients with an inflammatory condition but not cancer, such as IgA nephritis, did not show an increase in galectin-1 binding glycoproteins, but rather a decrease (not shown).

### Galectin-1 binds particular forms of a selected set of serum glycoproteins

**Figure 3:**
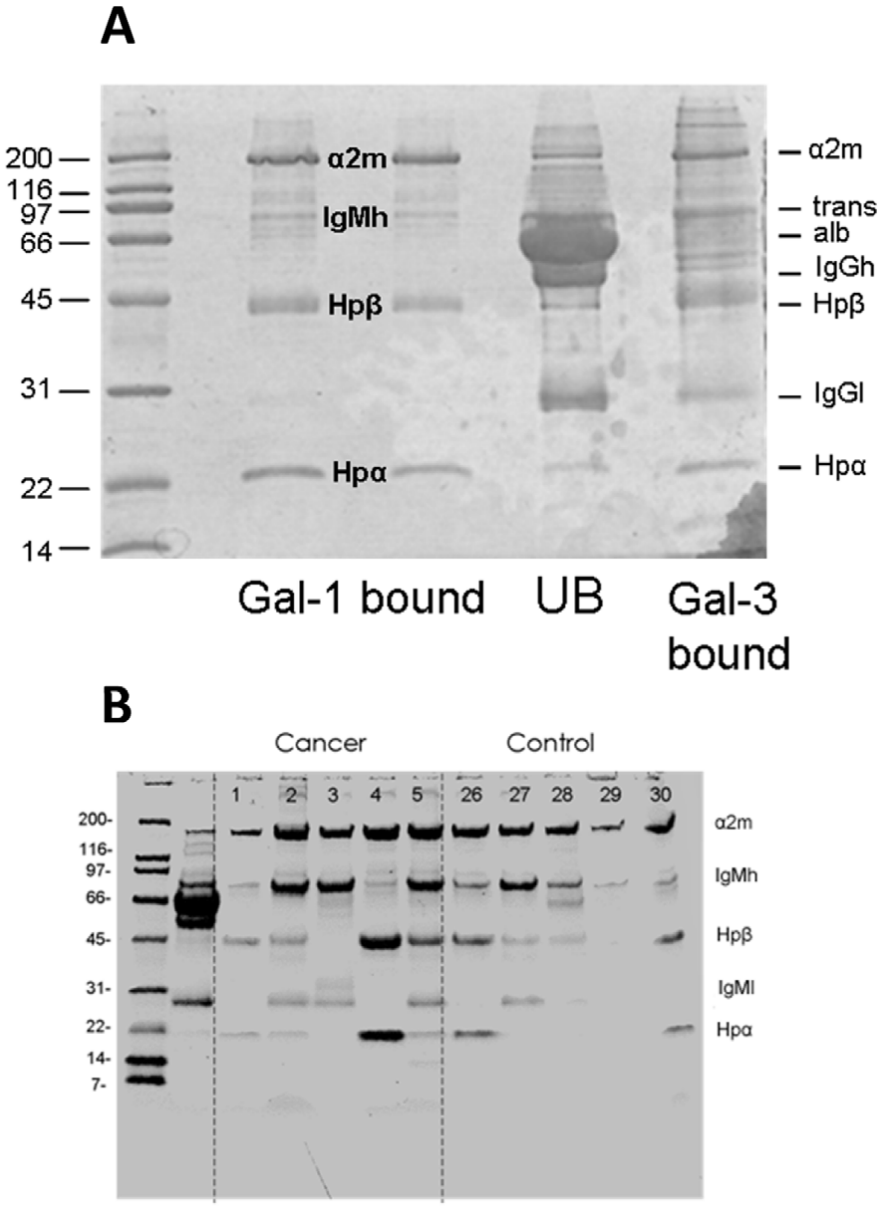
Identification of galectin-1 binding proteins in cancer and healthy sera. **A**) SDS-PAGE (4–20% stained with Coomassie) of bound fractions from one healthy individual separated with affinity chromatography on galectin-1 C3S (two lanes), the unbound fraction (lane UB), and bound fraction from the following affinity chromatography on galectin-3 (one lane). Indicated to the left are the mobilities of known size markers and to the right mobilities of the known serum proteins α-2 macroglobulin (α2 m), transferrin (trans), IgM heavy chain (IgMh), albumin (alb), IgG heavy chain (IgGh) and IgG light chain (IgGl), haptoglobin β chain (Hpβ) and haptoglobin α chain (Hpα). In between the lanes with galectin-1 binding fractions are indicated the proteins identified by MALDI-TOF-MS of tryptic digest of cut out bands. **B**) An example of SDS-PAGE of galectin-1 binding glycoproteins from 5 healthy and 5 cancer sera (one out of five gels). The highest binding fractions (from individuals numbered as in supportive material Table 1) were analyzed by SDS-PAGE (4–20% stained with Coomassie). Indicated to the left are the mobilities of known size markers and to the right known serum proteins abbreviated as for A. The second lane (unlabeled) shows unseparated serum from patient 1, with albumin as the major band.

Serum from a cancer patient was subjected to affinity chromatography on immobilized galectin-1 as described above. The unbound fraction was subsequently subjected to affinity chromatography on immobilized galectin-3, which binds a much larger fraction and wider range of human serum glycoproteins [25]. The major glycoproteins bound by either galectin were identified by MALDI-TOF mass spectrometry as indicated in Fig. 3A, and the identities were confirmed by western blotting (not shown). Galectin-1 bound mainly haptoglobin, IgM and α-2-macroglobulin. However, only a fraction of each of these glycoproteins were bound by galectin-1 as they were also found in the galectin-3 bound fraction derived from the galectin-1 unbound fraction. Thus, galectin-1 binds only particular forms of these proteins with the required affinity (K_d_∼1–5 µM or better). A similar result was found when serum from a healthy individual was applied to rat galectin-1, although less glycoproteins bound galectin-1 in this case [25].

**Figure 4:**
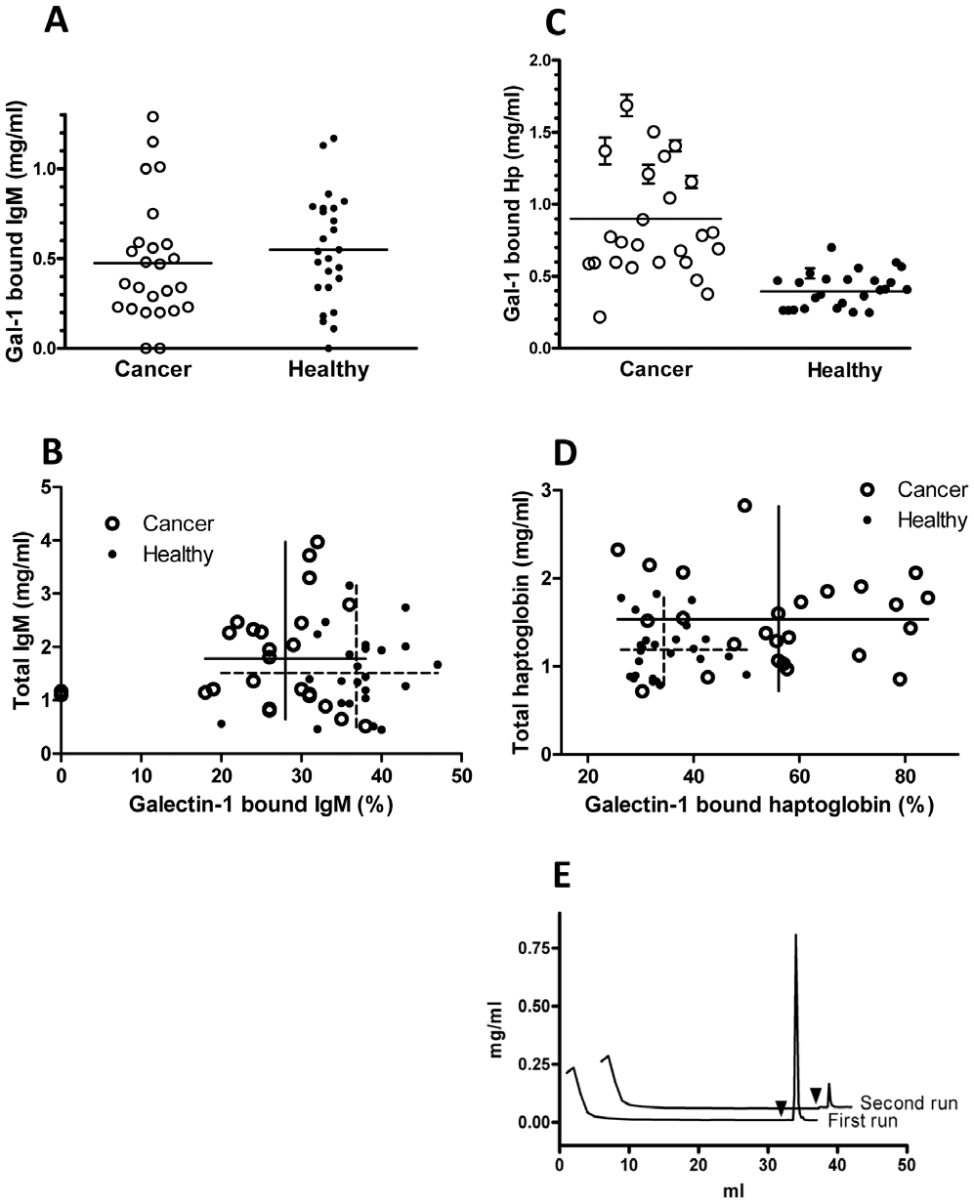
Quantitation of total and galectin-1 bound IgM and haptoglobin in cancer and healthy sera. **A**) The concentrations of IgM in the galectin-1 bound fractions from cancer (n = 25) and healthy sera (n = 25) as determined by direct enzyme-linked immunosorbent assay (ELISA). The average for each group is marked by horizontal lines, but the slight difference was statistically not significant (p = 0.41). **B**) Initial serum levels of IgM (Y-axis) compared to % of galectin-1 bound IgM (X-axis). The averages and ranges of each parameter are shown by crossed lines, unbroken for cancer sera and dashed for healthy sera. The difference between the % galectin-1 bound IgM was significant (p<0.0001) but the difference between total IgM was not (p = 0.24). The three samples with no galectin-1 bound IgM (seen as dots on Y axis) were excluded from the calculation of averages, ranges and significances shown; including them made only a minor change in averages and no change in significance of differences. **C**) The concentrations of haptoglobin in the galectin-1 bound fractions from cancer (n = 25) and healthy sera (n = 25) as determined by direct ELISA. The average for each group is marked by horizontal lines, and the difference was statistically significant (p<0.0001). Error bars represent SEM from three independent duplicate measurements for each data point. **D**) Initial serum levels of haptoglobin (Y-axis) compared to % of galectin-1 bound haptoglobin (X-axis). The averages and ranges of each parameter are shown by crossed lines, unbroken for cancer sera and dashed for healthy sera. The difference between the % galectin-1 bound haptoglobin was significant (p<0.0001) and the difference between total haptoglobin was also significant (p = 0.0058). **E**) Chromatograms of 2 mg purified pooled haptoglobin separated by affinity chromatography on columns (1 ml) with immobilized galectin-1 C3S. The protein concentration of each fraction (0.2 or 1 ml) is given on Y-axis. Elution with lactose (150 mM) started after washing with 32 ml PBS (arrow heads). Non-binding haptoglobin was chromatographed a second time but only traces (∼1%) of additional haptoglobin was now found in the bound fraction. The chromatogram from the second run has been moved for clarity by +0.05 on Y-axis and by +5 on X-axis.

The major galectin-1 bound glycoproteins in all 50 serum samples were α-2-macroglobulin, IgM and haptoglobin as confirmed by western blots (not shown), but with considerable variation in relative amounts between individuals, as exemplified in Fig. 3B and shown for haptoglobin in all samples in Fig. S1. To study these variations further, the concentrations of total and galectin-1 bound IgM and haptoglobin were determined by ELISA (Fig. 4).

### Galectin-1 binds a specific fraction of IgM which decreases slightly in cancer

The mean (1.5 mg/ml) and range (0.5–3.2 mg/ml) of IgM concentrations in the healthy sera were as expected, and slightly higher (not statistically significant) for the cancer sera (mean 1.8 mg/ml, range 0.7–4 mg/ml). The average concentration of galectin-1 bound IgM was slightly lower in cancer sera (not significant, Fig. 4A), due to a significant decrease (p<0.0005) in the percentage of IgM bound to galectin-1 (mean 37% for healthy sera and 28% for cancer sera) (Fig. 4B). In addition, and not included in this calculation, two cancer sera and one healthy serum did not contain any galectin-1 bound IgM (e.g. lane 4 and 29 in Fig. 3B, and dots on X-axis in Fig. 4A).

### Galectin-1 binds a specific fraction of haptoglobin which increases in cancer

The level of galectin-1 bound haptoglobin in the cancer sera was on average about twice as high (range 0.6–4 fold) compared to the average for control sera, (Fig. 4C) and confirmed by SDS-PAGE (Fig. S1). This was due to the binding of a larger fraction of haptoglobin (average 50%, range 20–80%) to galectin-1 (Fig. 4D, X-axis) for most cancer sera that had insignificant change of the total haptoglobin level (Fig. 4B, Y-axis), whereas for a few cancer sera, there was an increase in total haptoglobin level, but about the same fraction bound to galectin-1 as in control sera (average 30%, range 25–50%). Galectin-1 affinity chromatography of purified pooled human haptoglobin from healthy sera also resulted in a bound fraction of about 30% that could be eluted as a sharp peak using lactose (Fig. 4E), which was clearly distinct from the unbound fraction (about 70%) that flowed through the column without trailing, and gave less than 1% bound to the column when rechromatographed.

The known peptide chain polymorphism of haptoglobin [32] did not contribute to differences in galectin-1 binding, since SDS-PAGE of unseparated pooled haptoglobin, the galectin-1 unbound fraction, and the galectin-1 bound fraction, all showed the same proportion of the α^1^ chains, α^2^ chains, and β chains (Fig. S2). Moreover, all the three most common haptoglobin protein phenotypes (with one or both of the two α-chain variants) were represented among the 50 samples, in proportions similar to those known for the Swedish population [32] (not shown); hence, there is no evidence for preferential binding of any of these protein forms by galectin-1.

Instead, with galectin-1 being a carbohydrate binding protein, the data suggest that a subset of the different glycoforms of haptoglobin [2], [3] bind galectin-1 with high affinity, which can be clearly distinguished from other glycoforms with no or substantially lower affinity for galectin-1. Therefore, as next step we characterized these glycoforms and incorporated the N-glycans in known models of haptoglobin, to help inform on where galectin-1 might bind in relation to binding sites for other molecules on haptoglobin (Fig. 1)

### Characterization of galectin-1 bound and unbound haptoglobin glycoforms

**Figure 5:**
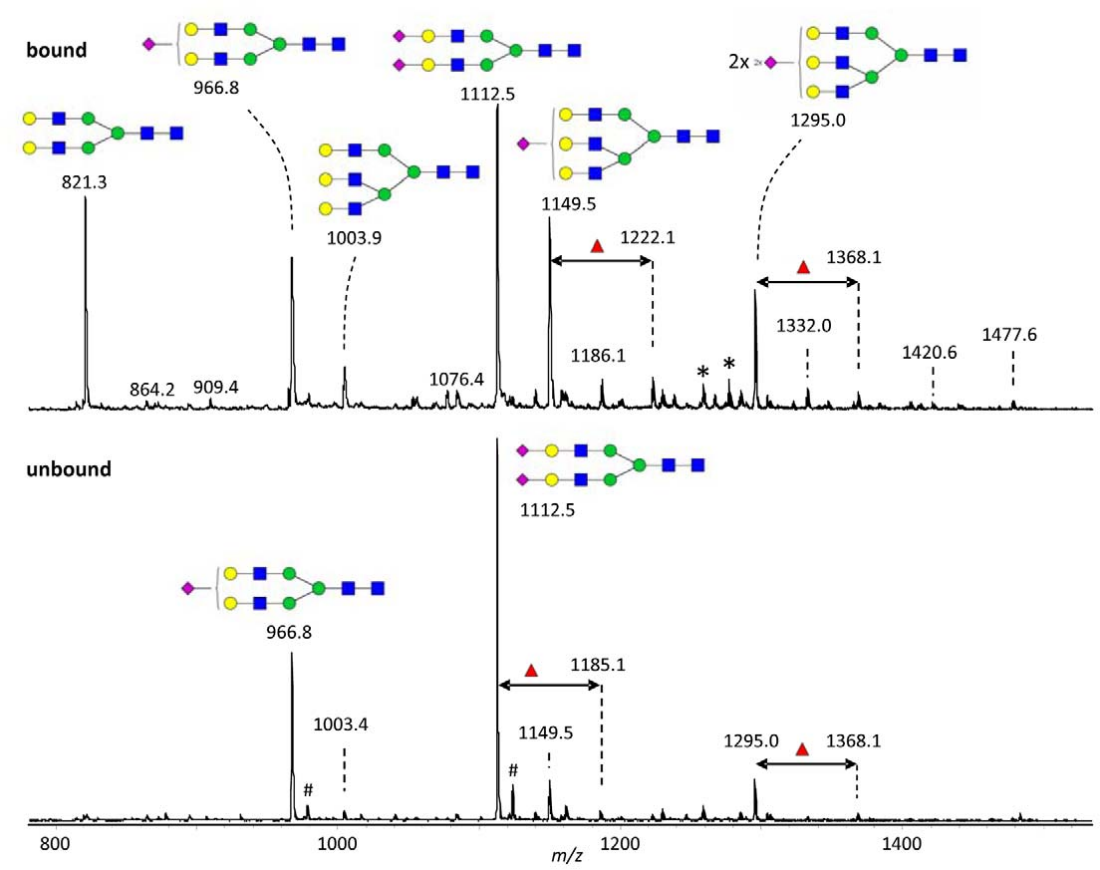
Mass spectrometric analysis of N-glycans from galectin-1 bound and unbound haptoglobin. Enzymatically released N-glycans from galectin-1 bound (top panel) and unbound (bottom panel) haptoglobin (pooled from human plasma, separated as described in S5) were analyzed by nano-LC-ESI-ion trap-MS. N-glycans were detected in double-protonated form giving m/z values about equal to half the molecular weight. Schematics of the glycans corresponding to the major peaks are shown, with symbols as described for Fig. 1C. Some minor peaks show glycans with an additional fucose residue (red triangle) as indicated by a double-headed arrow. Other minor peaks correspond to tetraantennary glycans with 0, 1 or 2 NeuAc residues (m/z1186.1, 1332.0 and 1477.6, respectively). #, natrium proton mixed adducts; *, single charged contaminants.

N-glycans were released from fractionated haptoglobin (galectin-1–unbound and –bound as in Fig. 4E) by PNGase F treatment and analyzed by graphitized carbon nanoLC-ion trap-MS/MS (Figure 5); glycan compositions and structural features were confirmed by tandem mass spectrometry (not shown). The unbound fraction had an N-glycan-profile resembling total serum glycoproteins, with a biantennary-disialoglycan as major species (m/z 1112.5), the monosialylated version (m/z 966.8) as second most predominant, and smaller amounts of triantennary and other N-glycans. The bound fraction had two major differences, an increased proportion of triantennary glycans (m/z 1149.5 and 1295.0), and an increased amount of a non-sialylated biantennary glycan (m/z 821.3). The combined profile (giving 30% weight to the bound fraction and 70% weight to the unbound fraction) is in good agreement with previous analysis of haptoglobin N-glycans, also including the presence of smaller amounts of fucosylated variants indicated by red triangles in Fig. 5 [2], [3].

Since haptoglobin has four known N-glycosylation sites (1–4 in Fig. 1A), we profiled the N-glycans at separate sites by analyzing tryptic peptides from galectin-1 bound and unbound haptoglobin, using reverse phase nanoLC-ion trap-MS (Fig. S3); glycopeptide assignments were confirmed by tandem mass spectrometry as exemplified in Figure S4. Two singly glycosylated peptides (site 1 and 4, Fig. S3A) and one doubly glycosylated peptide (site 2 and 3, Fig. S3B) were found, together covering the four known N-glycosylation sites of the haptoglobin β-subunit [2], [3], [33]; no evidence for other glycosylation sites was found. For the galectin-1 unbound haptoglobin, the glycosylation profile at each site resembled that of intact unbound haptoglobin (Fig. 5) with the predominant glycans being mono and disialylated biantennary species. For the bound fraction the N-glycan profile of site 1 and 4 were similar as found for the unbound fraction, but with slightly increased presence, as minor species, of the non-sialylated biantennary N-glycan (left panels in Fig. S3A) and triantennary glycans (right panels). The N-glycan profile of sites 2–3 (Fig. S3B) clearly differed, with presence of less sialylated glycans and much increased amount of triantennary glycans in the galectin-1 bound fraction. The profiles agree with Fig. 5 and the combined profiles (giving 30% weight to the bound fraction and 70% weight to the unbound fraction) again agree with previous publications of haptoglobin N-glycans, including the assignment of triantennary N-glycans mainly to site 3 [2]. These data suggest that interaction of haptoglobin with galectin-1 is not conferred by sites 1 and 4, but instead site 2 or 3 that are found in a region of the β-subunit away from the suggested binding sites for haemoglobin and uptake receptor CD163 (Fig. 1A).

### Evidence for a specific binding site for galectin-1 on haptoglobin

Previously we have shown that galectin-1 interacts with serum glycoproteins mainly in a monovalent fashion and that interaction with only one of its CRDs is sufficient for binding as defined here (that is binding to the affinity column requiring K_d_ in low µM range) [28]. Thus, interaction with only one N-glycan on a whole haptoglobin molecule (Fig. 1C) would be sufficient, which will be 1/8 of the total for a αβ dimer, 1/12 for a trimer and a smaller fraction for higher oligomers; with this calculation for pooled haptoglobin, as analyzed here, the galectin-1 binding N-glycan needs to be at most about 10% of the total N-glycans and at most about 30% of those at a specific N-glycosylation site.

LacNAc is by far the major β-galactoside on serum glycoprotein and is expected to be bound in the core binding site (C–D) of the galectin (the LacNAc residues shown in black in Fig. 1C). By itself, however, it binds galectin-1 only with a K_d_ of 100–200 µM [28], [34], which is too weak to confer the binding observed here. The potency of total haptoglobin (counted as the dimer) as an inhibitor in the fluorescence anisotropy assay corresponded to an average K_d_ of ∼2.5 µM (Table S2), and thus for the 30% bound fraction estimated to be less than 1 µM. Therefore some additional interactions must be required. Making variants of natural serum haptoglobin, with altered glycosylation and/or peptide sequence, to identify these interactions is very difficult or impossible at the present time. Instead we made mutants of galectin-1 to identify what aspects of its binding properties are required for its binding to serum glycoproteins and haptoglobin (Table S2 and Fig. S5).

One set of mutants were made in site B of the galectin (see Refs [20], [28] for detailed description of the 5 subsites (A–E) of the galectin carbohydrate binding groove), to alter interactions of the galectin with moieties linked to the 3-position of the Gal in LacNAc, in analogy with mutants previously made of galectin-3 [28]. Galectin-1 binds terminal LacNAc residues and those carrying sialic acid [35] or sulphate [36] at the 3-position of Gal, but in contrast to galectin-3 it does not tolerate other extensions, e.g. by GlcNAcβ as found in polylactosamines (represented by GlcNAcμ1-3Lac in Table S2). Two mutants, N34D and S30G had selectively reduced tolerance for 3-sialylated galactosides, but bound terminal LacNAc with about equal affinity as galectin-1 C3S (Table S2). These mutants also bound less serum glycoprotein and haptoglobin, in rough proportion to their loss of tolerance for sialylation, suggesting that the main high affinity binding site for galectin-1 includes a 3-sialylated galactoside. To determine if 3-linked sialic acid was required for binding or only needed to be tolerated, neuraminidase treated serum was analyzed. This treatment restored binding of serum glycoproteins to the N34D mutant to a level similar to wild type galectin-1. Thus, the 2–3 sialic acid must be tolerated but is not required for binding.

2–3 sialylated galactosides have been found only in triantennary N-glycans in human serum (e.g. like structure 3 of Fig. 1B), and in haptoglobin predominantly at site 3 [2], [3], [37]. The intensity of the peaks for triantennary glycans in the mass spectra of the galectin-1 bound haptoglobin N-glycans (Fig. 5 and Fig. S3), even if only semiquantitative, suggest that their percentages of the total are sufficient to account for the galectin binding (>10% of the total and >30% at site 3). 3-O sulfated galactosides have been shown to bind galectin-1 with enhanced affinity [36], [38] but they would have been detected by the mass spectrometry employed here, and hence, are not likely candidates as galectin-1 binding sites on haptoglobin.

Another site B mutant of galectin-1, V32A, opens the ability to bind GlcNAcβ1-3 substituted galactosides (Table S2), as found in internal Gal-residues of polylactosamines (repeating LacNAc residues). This does not appear to be important for binding to serum glycoproteins, as this mutant bound the same amount and profile of glycoproteins as galectin-1 C3S. Previous studies have suggested high affinity (K_d_ in µM range or better) binding of galectin-1 to polylactosamines, but later studies have shown that galectin-1 only binds terminal LacNAc residues, and the apparent preference for polylactosamines in some assays is to put these far enough out [39], [40]; in fact polylactosamines do not bind galectin-1 better than the ordinary types of N-glycans shown here on haptoglobin [27], [41]. Polylactosamines have not been reported among human serum N-glycans despite studies by many groups, making them unlikely binding sites for galectin-1 in the present study. Nevertheless, to further assess their role for the binding of galectin-1 to serum glycoproteins, we passed a serum sample through a column with immobilized tomato-lectin, known to bind polylactosamines selectively [40], and then analyzed the flow through on immobilized galectin-1 as described above. There was no significant reduction in recovery of galectin-1 bound glycoproteins, suggesting that most of the binding to galectin-1 to serum glycoproteins is not mediated by polylactosamines. In addition no serum proteins were found when a sample from the top of the tomato-lectin column was boiled in sample buffer and analyzed by SDS-PAGE (not shown).

One mutant in site E, R74S, near the reducing end of LacNAc in site C–D, was also tested. This mutant was harder to produce and, therefore, not tested in affinity chromatography. However, in inhibition assays it was clear that it had dramatically reduced (>8 fold) affinity for haptoglobin even if its affinity for LacNAc was identical to wild type galectin-1 (Table S2). This strongly suggests that for high affinity binding, galectin-1 also has to interact with parts of the N-glycan near the reducing end of LacNAc or with nearby protein parts.

The non-sialylated biantennary N-glycan (#1 in Fig. 1B) was also enriched in the galectin-1 bound haptoglobin fraction and present in sufficient amount to be a galectin-1 binding site. However, by itself it is a weak ligand for galectin-1 (K_d_ about 100 µM, [28]), and less than 2% of neuraminidase treated transferrin that carries almost only this glycan bound galectin-1 (Fig. S6). Thus, together with the data presented above, this strongly suggests that a major recognition site for galectin-1 on haptoglobin is a triantennary N-glycan, such as #3 of Fig. 1B, but binding to other glycans (e.g. #1 and 2) cannot be ruled out if they are presented in a particularly favorable way in conjunction with the protein.

High affinity binding of galectin-1 could also, theoretically, be caused by interaction with multiple available binding sites on the same glycoprotein molecule, where high affinity is either caused by simultaneous binding of a galectin-1 dimer at two sites, or by a recapture effect [34]. However, our previous studies argued against these mechanisms [28], e.g. showed that a dimerization deficient mutant of galectin-1 bound serum glycoproteins with equal affinity as wild type, and instead as mentioned above, showed that galectin-1 interaction with glycoproteins is largely monovalent. Moreover, divalent binding of a galectin-1 dimer to two N-glycans within the same haptoglobin subunit is not possible because the galectin-1 carbohydrate binding sites are too far apart and have opposite directions (black surfaces in yellow model in Fig. 1C). Divalent binding between two subunits of a haptoglobin multimer is also unlikely as galectin-1 would not reach far enough. From the above we conclude that one specific site on a haptoglobin molecule interacts with one CRD of the galectin-1 dimer, leaving the other open for interaction with cellular glycoproteins involved in the function of haptoglobin. This is not likely to be functionally significant in serum or plasma itself since there the concentration of galectin-1 dimer is very low (<1 nM [42], <3 nM (not detected) as confirmed here for 5 of the healthy and cancer samples. Instead we tested the possibility that interaction with galectin-1 in cellular compartments, where its concentration may be much higher, is important for the function of haptoglobin.

### Different intracellular localization of galectin-1 binding and non-binding haptoglobin after endocytosis in macrophages

The major function of haptoglobin is to capture free haemoglobin (Hb) released in the circulation during haemolysis, resulting in clearance of potentially oxidative and toxic iron groups that haemoglobin contains [43]. The Hp-Hb complex can then be internalized through binding of haptoglobin to CD163, a scavenger receptor expressed on the surface of cells from the macrophage lineage [44], in particular in alternatively activated anti-inflammatory macrophages [45], [46].

In order to study the relationship between these processes and the galectin-1 binding of haptoglobin, we made complexes of haemoglobin with galectin-1 unbound and bound haptoglobin (Fig. S7A), and studied their uptake into THP-1 cells, differentiated with PMA and alternatively activated by IL-4 as a macrophage model [47]. The PMA treated THP-1 cells became adherent as expected, and after the additional IL-4 activation they became larger and more granular (Fig. S7B) and showed specific staining for CD163, albeit sparse (not shown).

The cells were functionally characterized by incubation with 0.2 µM rhodamine labeled haptoglobin, haemoglobin, or Hp-Hb complex (galectin-1 non-bound) followed by epifluorescence microscopy to follow uptake. Haptoglobin was not internalized after 30 minutes in any of the THP-1 cells tested, and also not in a number of other galectin-1 expressing cell lines (CHO, HFL-1, SkBr3) (not shown). Treatment of cells with 0.2 µM haemoglobin for 30 minutes, as expected, resulted in cell death (cell shrinkage, nuclear fragmentation and detachment). In contrast, the Hp-Hb complex was internalized in the PMA+IL-4 stimulated THP-1 cells after 30 min, but not in the cells stimulated with PMA only (Fig. S7C). Galectin-1 was found in the cytosol and nuclei as known for other cell types but also in larger granules, and its staining pattern was more distinctly vesicular and “patchy” in the IL-4 stimulated THP-1 cells (not shown), similar to Jurkat T-lymphocyte cells as previously described by Fajka-Boja *et al.* [48].

**Figure 6:**
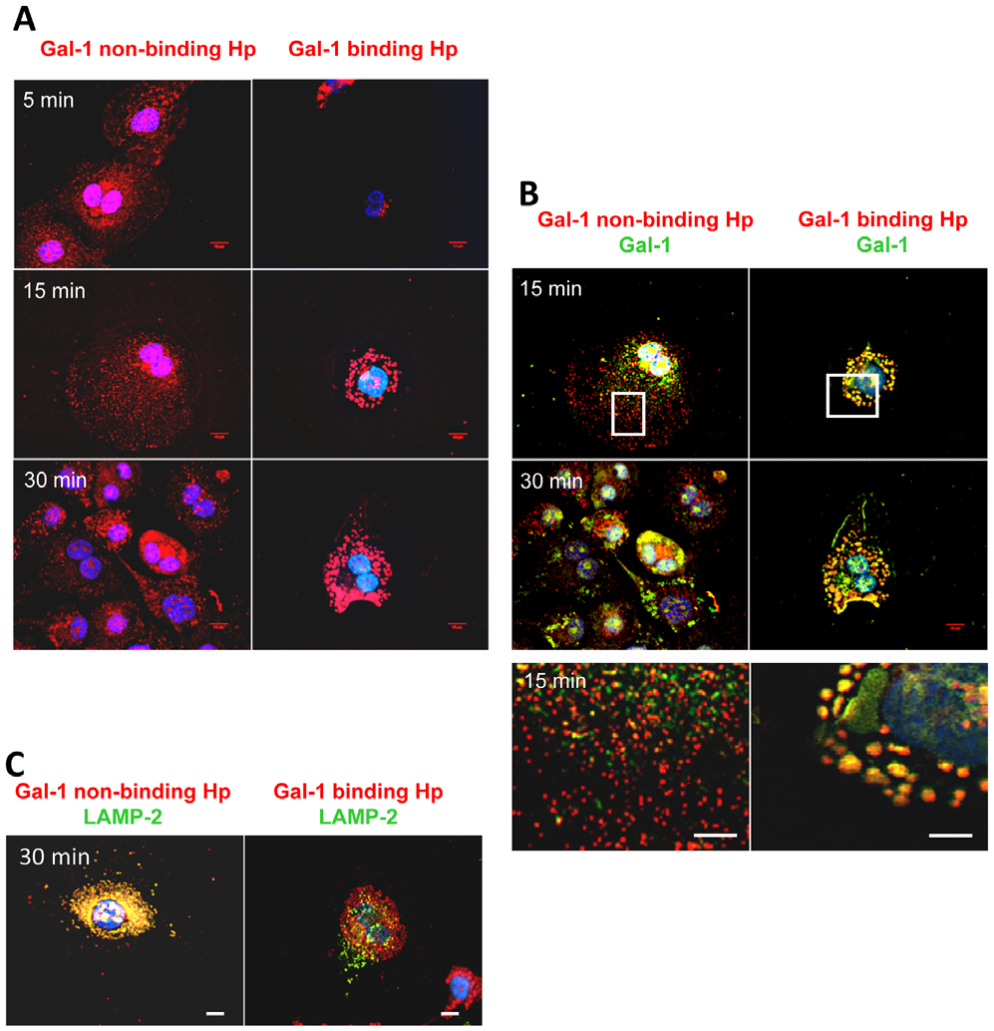
Endocytosis and intracellular trafficking of galectin-1 unbound and bound haptoglobin-haemoglobin complex in macrophages. **A**) THP-1 cells treated to differentiate with PMA for 5 days, and alternatively activated with Il-4 for 2 days were incubated with 0.2 µM rhodamine-labeled galectin-1-non-binding (left panel) or -binding (right panel) haptoglobin (pooled from human plasma, separated as described in Fig. S5) in complex with haemoglobin (red) for 5, 15 or 30 minutes at 37°C in order to allow endocytosis to occur. Cells were fixed in formaldehyde, nuclei stained blue with Hoechst, and cells observed by deconvolution fluorescence microscopy (100×). Scale bar represents 10 µm. **B**) Images from Panel A in combination with immunostaining for galectin-1 (green). Extensive colocalization of galectin-1-binding haptoglobin-haemoglobin and galectin-1 is revealed by the overlap of signals in merged images that results in yellow staining. The bottom panels show a larger magnification of the boxed areas from the top panels. Scale bar represents 10 µm. **C**) Cells were treated as for panels A and B except that they were immunostained for LAMP-2 (instead of galectin-1), a marker for late endosomes and lysosomes. Extensive colocalization of galectin-1-non-binding haptoglobin-haemoglobin and LAMP-2 is revealed as yellow staining.

Rhodamine labeled galectin-1 binding or non-binding Hp-Hb-complexes (0.2 µM) were added to the PMA+IL-4 stimulated THP-1 cells and the intracellular localization was analyzed after 5, 15 and 30 min by deconvolution fluorescence microscopy. The galectin-1 binding Hp-Hb-complex had a completely different intracellular distribution compared to the non-binding Hp-Hb (Fig. 6A) already after 5 min, and even more pronounced after 15 and 30 min. The galectin-1 binding Hp-Hb-complex was found in large granule like vesicles, which were also stained by an anti-galectin-1 antibody (green in Fig. 6B). In contrast, the galectin-1 non-binding complexes did not colocalize with galectin-1, but instead with LAMP-2 (green in Fig. 6C), a marker for late endosomes and lysosomes [49]. The colocalization was quantitated using the overlap coefficient of Manders, which gives the true degree of colocalization (e.g. Manders coefficient 0.5 implies that 50% of the two selected channels co-localize) [50]. As expected, the added galectin-1 binding Hp-Hb-complex showed a significant overlap (Manders coefficient; 15 min: 0.81 and 30 min: 0.79) with endogenous galectin-1 (Fig. 6B), whereas non-binding Hp-Hb-complex showed hardly any overlap with galectin-1 (Manders coefficient; 15 min: 0.47 and 30 min: 0.42). In contrast, staining with LAMP-2 antibody showed a considerable overlap with non-binding Hp-Hb-complex (Manders coefficient 0.91) but not with galectin-1 binding Hp-Hb-complex (Manders coefficient 0.29) (Fig. 6C). Thus, two subpopulations of human haptoglobin glycoforms, separated based on their binding to galectin-1, form complexes with Hb that are subsequently sorted to different endocytic compartments in macrophages.

## Discussion

The present results demonstrate that a significant fraction of human serum α-2-macroglobulin, IgM and haptoglobin, bind to galectin-1 and that the total amount of galectin-1-binding glycoproteins can be 2–3 times larger in sera of some cancer patients. If albumin and IgG, both of which do not bind galectin-1, are excluded, galectin-1 binds approximately 10–15% of the remaining glycoproteins in healthy sera and up to 40% in cancer sera. This dramatic increase raises three questions: 1) what is the mechanism, 2) what is the functional consequence, and 3) what is the diagnostic value?

The mechanism was studied in detail for haptoglobin. The composition of N-glycans of galectin-1 bound and unbound haptoglobin was analyzed (Fig. 5 and S3) and incorporated into models of haptoglobin (Fig. 1). Together with previous knowledge that the interaction with galectin-1 is mainly monovalent [28], the data suggest that interaction of one galectin-1 CRD with one N-glycan on haptoglobin is sufficient for binding. This interaction must involve some special feature of the N-glycan and probably also neighboring parts of the protein. The loss of binding of the N34D mutant suggests that the binding site includes a 2–3 sialylated galactose residue, such as has been found on the third antenna of triantennary N-glycans in serum and haptoglobin (structure in Fig. 1), but additional interactions are most likely also required to reach the affinity observed. Since each haptoglobin molecule contains 8 or more N-glycans (Fig. 1C), the galectin-1 binding glycan accounts for only 10% or less of these, and therefore other N-glycans, not binding galectin-1, are carried along in the galectin-1 bound fraction. These other glycans may also be part of a cancer related glycan profile, if, for example, the haptoglobin comes from cancer cells, as proposed [51], or liver cells indirectly affected by the presence of cancer elsewhere in the body. Therefore, previous studies and the present study may have detected overlapping sets of cancer related haptoglobin glycoforms. For example, the fucosylated N-glycans, albeit quantitatively minor, that were found in increased amounts on haptoglobin from different cancer patient sera [2], [3], were also found enriched in the galectin-1 bound fraction (indicated by red triangles in Fig. 5), even if such antennary fucose (linked to 3 position of GlcNAc) would block galectin-1 binding [20].

The proposed one site mechanism for galectin-1 binding to haptoglobin may also explain binding to IgM and α2-macroglobulin, although further studies like the ones applied here to haptoglobin are required to confirm this. Each IgM pentamer has at least 51 N-glycosylation sites (5 conserved for each of the 10 heavy chains +1 for the J-chain) [52]. Thus an N-glycan conferring high affinity galectin-1 binding to IgM needs to be present only as <1% to explain the binding of about 30% IgM as found here. Therefore, triantennary complex N-glycans may explain binding of galectin-1 also to IgM even if they occur only as very minor species [52]. By an analogues argument, the binding of a major part of α2-macroglobulin to galectin-1 can also be explained; this tetrameric glycoprotein carries 32 N-glycans (8 per subunit) and triantennary complex N-glycans are well above the 3% (one per molecule) required for galectin-1 binding [53]. While there is an increasing number of reports suggesting changes in O-linked glycosylation as the major cancer-associated post-translational modification [54], this will most likely not be the source of increased galectin-1 binding of serum glycoproteins. First, none of the three galectin-1 bound glycoproteins carry any known O-glycan, while their N-glycosylation is well characterized. Second, IgA, the major O-glycosylated protein in serum is not bound by galectin-1 [25], and third the major cancer associated O-glycans, the T antigen (Galβ1-3GalNAcα) and Tn-antigen (GalNAcα) are poor ligands to human galectin-1[35], [55].

The binding of one galectin-1 CRD to haptoglobin leaves the other available to cross-link it to other glycoproteins in the tissue cell, which we believe is the function of this interaction. The interaction is not likely to be significant in serum itself, because there the measured concentration of galectin-1 dimer is <1 nM [42] compared to >1 µM, i.e. more than 1000 fold more, for either of the main bound glycoproteins. After exit from the blood stream, however, serum glycoproteins will encounter high concentrations of galectins (µM) in tissue cells, with possible mutual functional effects as discussed by Cederfur *et al.* [25]. Here we show that the interaction of haptoglobin with galectin-1 becomes decisive after haptoglobin has carried out its highly specialized function – to form a tight complex with haemoglobin that binds to the scavenger receptor CD163 in alternatively activated macrophages and becomes endocytosed [43]. At this point galectin-1 binding appears to divert the complex from targeting into late endosomes and lysosomes, perhaps for degradation, and instead targets it to another set of larger vesicles for an as yet unknown function (Fig. 6). On average 30% of haptoglobin from healthy sera take this route, whereas for cancer sera it may be up to 80%. This suggests that this is a major regulatory step in the scavenging of haemoglobin by haptoglobin, and that this regulation can be dramatically altered in cancer.

Macrophages are abundant in breast carcinoma and reports suggest that these TAMs (tumour associated macrophages) exhibit an alternatively activated (MΦ2-like) functional profile and are CD163 positive [56], [57]. Thus one may speculate what would be the effect of the galectin-1 regulated balance between the different fates of Hp-Hb within these macrophages. For example, if the binding of haptoglobin to galectin-1 increases, more of the complex could end up in galectin-1 containing granules instead of being directed to the degradation pathway, which could influence haptoglobin and haemoglobin activities such as angiogenesis [58].

The other galectin-1 binding proteins were not analyzed in detail in this study, but a few points may still be made. Total IgM was slightly higher in cancer sera but the percentage bound to galectin-1 lower. This suggests that the IgM induced under the cancer condition may have decreased binding to galectin-1 with possible connection to its origin and function in the immune system. Another interesting finding was the three cases (two cancer sera and one healthy serum) with no galectin-1 binding IgM. There was no apparent correlation with the total amount of IgM or galectin-1 bound or unbound fraction of the other glycoproteins. These “outliers” may instead be the result of genetic polymorphisms, perhaps affecting N-glycosylation of IgM or a particular glycosyltransferase in B-cells. With the recent emergence of large scale profiling of N-glycans in human serum, genetic variants have begun to be discovered in the normal population [5], [59].

To address the final question on the diagnostic usefulness of galectin-1 binding serum glycoproteins, further studies are needed. These needs to include a larger number of cases, and cases of different stages of cancer development, especially early. This should also include comparison with sera from patients with benign breast disease. However, the purpose here was only to analyze if there was any difference between sera from patients with severe cancer and healthy controls (selected to be as similar to the patients as possible) at all. Already now it is clear that the quantitation of galectin-1 binding glycoforms is able to discriminate sera from patients with established metastatic cancer, as studied here, from sera from healthy controls. For the concentration of galectin-1 bound haptoglobin as the measured parameter, ROC plots (Fig. S8) gave an area under the curve (AUC) of about 0.90. This was also the case for percentage of galectin-1 bound IgM, and a combination (ratio) of the two parameters gave an AUC of 0.95. These values being >0.90 are considered excellent in the field, and are equally good or better than other proposed biomarkers for cancer in serum [8], [9], [26], [60].

Thus, further study of galectin-binding glycoforms of serum proteins is likely to be highly fruitful, but has been studied to a very limited extent so far. One study found strongly increased binding of galectin-3 to a haptoglobin like protein on western blots of sera from cancer patients, but only after desialylation [51]. Other earlier studies have found variable correlation between cancer and serum levels (low µg/ml) of the Mac-2-binding protein, named based on its affinity for galectin-3 [61]. Currently there is a great need for improved diagnostic biomarkers for breast and prostate cancer. Even one of the best markers established, PSA in prostate cancer, has significant overlap between non-cancer and cancer cases and due to its low sensitivity and specificity (area under ROC-curve typically ∼0.7, [62]) it is not widely used for population screening [63]. Most tumour markers currently used in clinics are serum glycoproteins (PSA, CA-125 and CEA); although usually the protein itself is detected, some tumour associated antigens consist of attached carbohydrates. In the evolving field of cancer biomarkers discoveries, detection of glycosylation changes is considered to be the frontline of future diagnostics, since they are capable of increasing sensitivity and specificity of existing protein-based assays [8], [64]. The studies here provide a novel approach because firstly, the cancer related glycoforms are detected using an endogenous lectin (galectin-1) that they are likely to interact with *in vivo*, and secondly because we show that this interaction results in a different function relevant for cancer. Thirdly, these functionally different glycoforms may make up a large proportion of major serum glycoproteins such as haptoglobin (up to 80% in some cases), making it likely that they reflect a changed physiological state of the patient.

## Supporting Information

Figure S1 **Quantitation of galectin-1 C3S bound haptoglobin in cancer and healthy sera.** (A) Intensities of haptoglobin heavy chain bands on SDS-PAGE gels (shown cropped) were estimated using histogram data from ImageJ (National Institutes of Health) using one of the known size markers as an internal control for each gel. Intensities are given in arbitrary units (AU) under each band, and sample number is given above it, with #1–25 from cancer patients and #26–50 from healthy controls (Table 1). (B) Haptoglobin band intensities (arbitrary units) for cancer patients and healthy controls. The average for each group is marked by horizontal lines.
(TIF)

Figure S2 **SDS-PAGE of haptoglobin from pooled healthy sera separated on immobilized galectin-1 C3S.** Unseparated (US), galectin-1 C3S unbound (UB1 circulated fraction, UB2 wash fraction) or bound (B) haptoglobin were analyzed by SDS-PAGE (4–20% stained with Coomassie). Indicated to the left are the mobilities of known size markers and to the right the different haptoglobin chains.
(TIF)

Figure S3 **A. Glycosylation analysis of site 1 (N59) or 4 (N116) of galectin-1 C3S bound and unbound haptoglobin.** Tryptic glycopeptides were identified by nano-LC-ESI-ion trap-MS, and MS peaks are shown for those carrying the six major N-glycans (schematics above with symbols as in Fig. 5). For site 1 (N59) they were detected as double peaks due to partial oxidization (ox) of methionine giving a 16 Da mass shift. **B. Glycosylation analysis of site 2 (N59) and 3 (N116) of galectin-1 C3S bound and unbound haptoglobin.** Tryptic glycopeptides containing the two sites were identified by nano-LC-ESI-ion trap-MS, and MS peaks are shown for those carrying the major detected combinations of N-glycans (schematics above mass spectra).
(PDF)

Figure S4 **Tandem mass spectrometry of glycopeptide M54-K77 carrying a biantennary, non-sialylated N-glycan.** The quadruple protonated glycopeptide was subjected to ion trap-MS/MS analysis.
(TIF)

Figure S5 **Affinity chromatography of serum and haptoglobin on galectin-1 C3S mutants.** (A) Serum from a healthy individual was analyzed using galectin-1 C3S and three further mutants in site B. Only the lactose eluted fractions are shown. Conditions are identical as in Fig. 2. (B). SDS-PAGE of peak bound (BF) and unbound (N-BF) fractions from the experiment of (A). The unfractionated serum (St.fr.) and size markers are shown to the left. All bound fractions showed the same pattern of proteins with α-2-macroglobulin, IgM and haptoglobin as major species, except that mutant N34D bound less of all, most clearly visible for haptoglobin. (C) Affinity chromatography on galectin-1 C3S and C3S N34D of serum treated or not treated with *Vibrio cholerae* neuraminidase. N34D binds much less untreated serum glycoproteins, but neuraminidase restores binding to equal levels as for galectin-1 C3S. (D) Affinity chromatography of haptoglobin (from pooled human plasma (Sigma-Aldrich)) on galectin-1 C3S and C3S N34D.
(TIF)

Figure S6 **Human transferrin analyzed by affinity chromatography on galectin-1 C3S or galectin-3.** 2 mg of human transferrin were analyzed using galectin-1 and galectin-3 coupled 1 ml affinity columns. As predicted galectin-1 does not bind human transferrin, while approximately 6% bind galectin-3 (top panel). The galectin-3 non-binding transferrin was treated or not treated with *Vibrio cholerae* neuraminidase (NA) (0.1 µmol 1 h at 37°C), and again analyzed on galectin-3 or galectin-1 coupled affinity columns. Removal of sialylations generated an additional 4% of galectin-3 binding transferrin (middle panel), but only traces (about 1.5%) of galectin-1 binding transferrin (bottom panel).
(TIF)

Figure S7 **Analysis of haptoglobin-haemoglobin complex in macrophages.** **A**) Native gel electrophoresis of haptoglobin-haemoglobin complex. Haptoglobin (left) moved into the gel, while haemoglobin (middle) did not. For the haptoglobin-haemoglobin complex (right) the free haptoglobin band had disappeared from the gel, indicating that the complex had formed. **B**) Light microscope images (40×) of THP-1 cells, untreated, induced to differentiate with PMA (5 days), or in addition IL-4 (2 days) for alternatively activation. Untreated cells were transferred to a polylysine glass slide and allowed to sediment for 15 min at room temperature before microscopy, while treated cells were grown directly on coverslips. Scale bar represents 50 µm. **C**) Uptake of Hp-Hb complex in differentiated and activated THP-1 cells. THP-1 grown in the presence of PMA (5 days) or PMA (5 days) +IL-4 (2 days) were incubated with 0.2 µm NHS-sulphorhodamine conjugated galectin-1 non-binding haptoglobin in complex with haemoglobin for 30 minutes. Cells were fixed in formaldehyde and analyzed by fluorescence microscopy (40×). Nuclei were stained blue with Hoechst. Scale bar represents 50 µm. All microscopy images were taken with a Nikon Eclipse TE2000-E fluorescence microscope.
(TIF)

Figure S8 **Receiver operating characteristic (ROC) curve analysis for different measured parameters to distinguish sera from breast cancer patients from controls.** A) Concentration of galectin-1 bound haptoglobin, B) percentage of galectin-1 bound IgM, and C) ratio of the percentages of galectin-1 bound haptoglobin and IgM. The area under the curve (AUC) indicates the discriminatory power of the measured parameter. A value >0.90 is considered excellent.
(TIF)

Table S1 **Subjects and respective amount of galectin-1 ligands in sera.**
(TIF)

Table S2 **Relative affinity of galectin-1 proteins for small saccharides and haptoglobin.**
(DOCX)
