# Supplementary figures and images for: Galectin-1-Binding Glycoforms of Haptoglobin with Altered Intracellular Trafficking, and Increase in Metastatic Breast Cancer Patients
Source: PLoS One. 2011 Oct 18;6(10):e26560. doi: 10.1371/journal.pone.0026560 (PMC3196588; doi:10.1371/journal.pone.0026560)

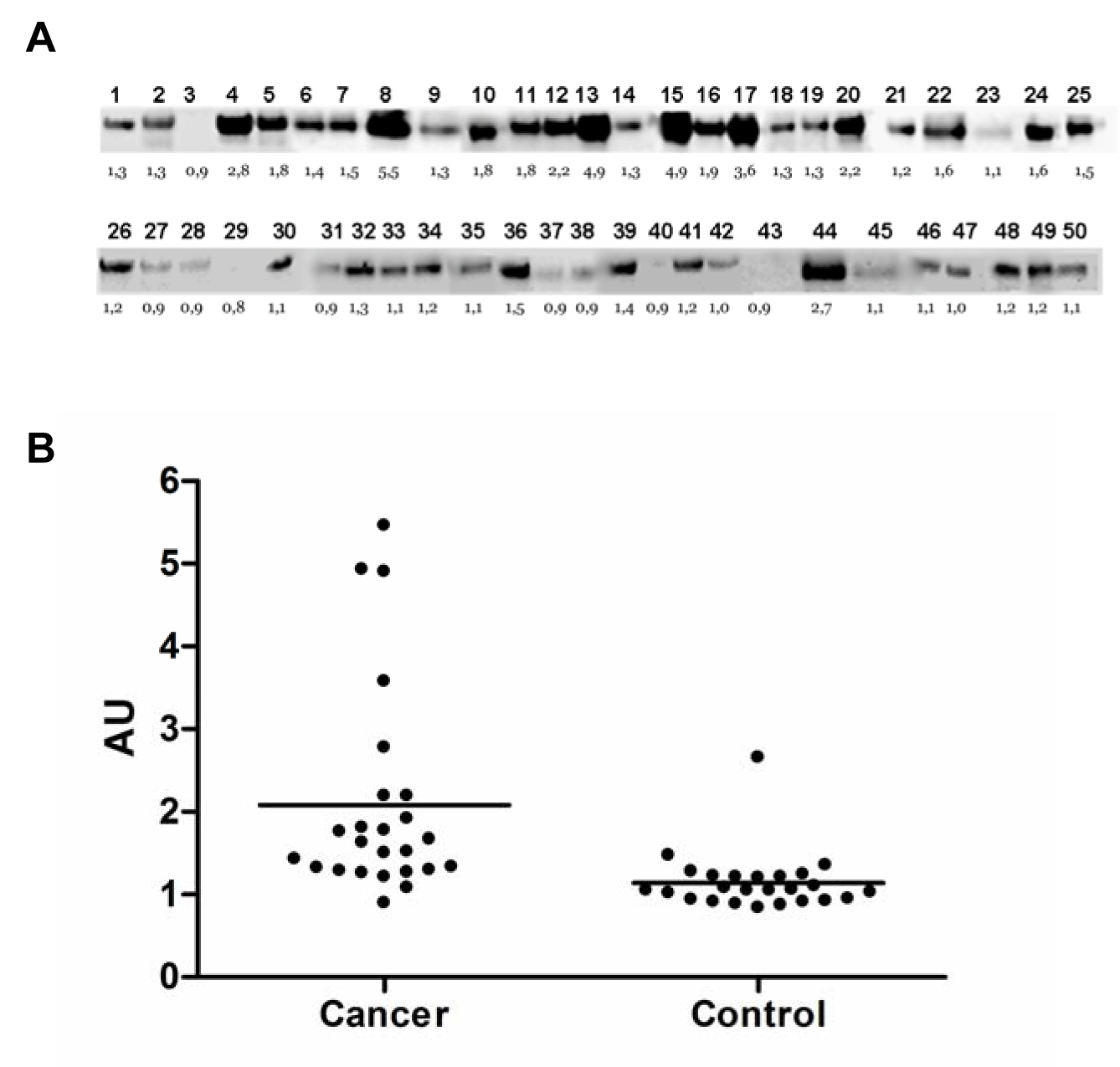

Supplement: Figure S1 — Quantitation of galectin-1 C3S bound haptoglobin in cancer and healthy sera. (A) Intensities of haptoglobin heavy chain bands on SDS-PAGE gels (shown cropped) were estimated using histogram data from ImageJ (National Institutes of Health) using one of the known size markers as an internal control for each gel. Intensities are given in arbitrary units (AU) under each band, and sample number is given above it, with #1–25 from cancer patients and #26–50 from healthy controls (Table 1). (B) Haptoglobin band intensities (arbitrary units) for cancer patients and healthy controls. The average for each group is marked by horizontal lines. (TIF) [file pone.0026560.s001.tif]

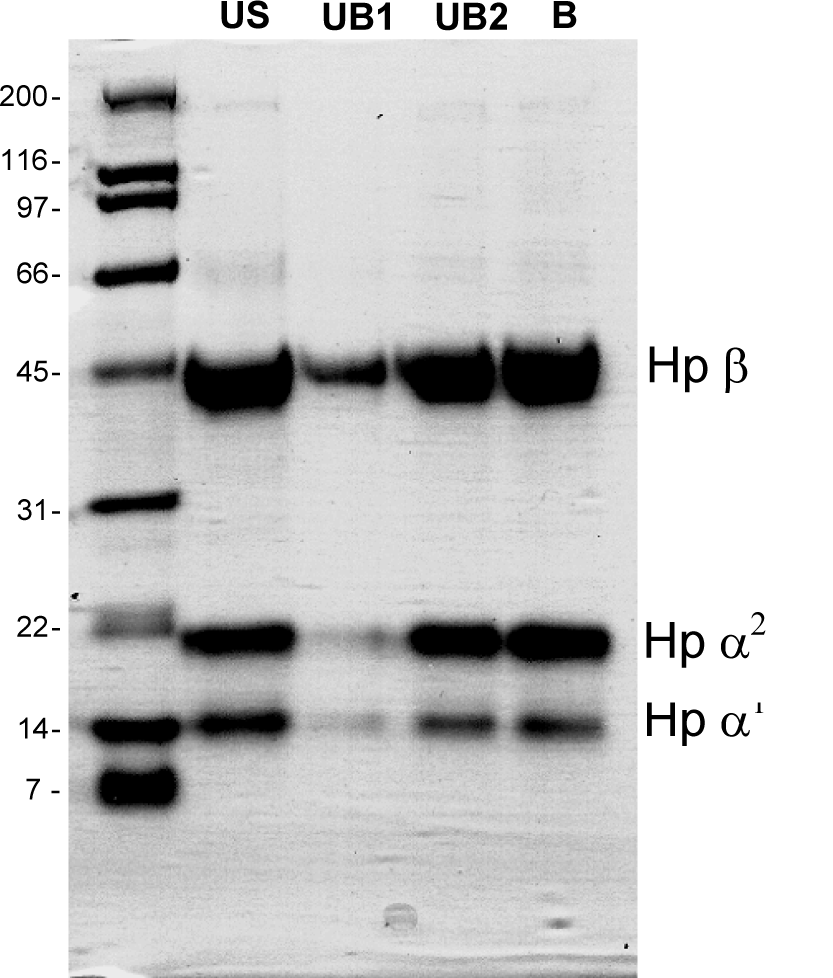

Supplement: Figure S2 — SDS-PAGE of haptoglobin from pooled healthy sera separated on immobilized galectin-1 C3S. Unseparated (US), galectin-1 C3S unbound (UB1 circulated fraction, UB2 wash fraction) or bound (B) haptoglobin were analyzed by SDS-PAGE (4–20% stained with Coomassie). Indicated to the left are the mobilities of known size markers and to the right the different haptoglobin chains. (TIF) [file pone.0026560.s002.tif]

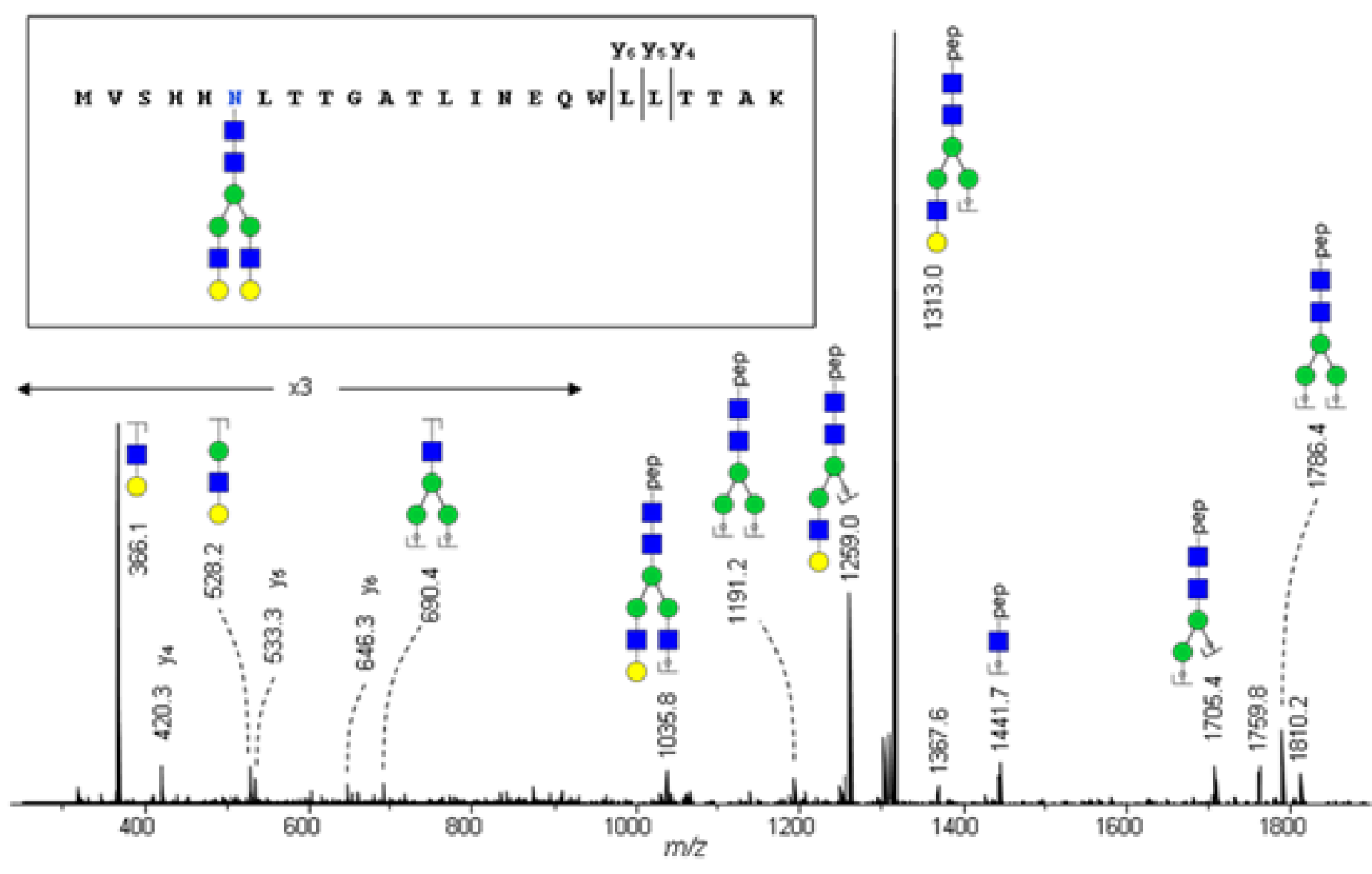

Supplement: Figure S4 — Tandem mass spectrometry of glycopeptide M54-K77 carrying a biantennary, non-sialylated N-glycan. The quadruple protonated glycopeptide was subjected to ion trap-MS/MS analysis. (TIF) [file pone.0026560.s004.tif]

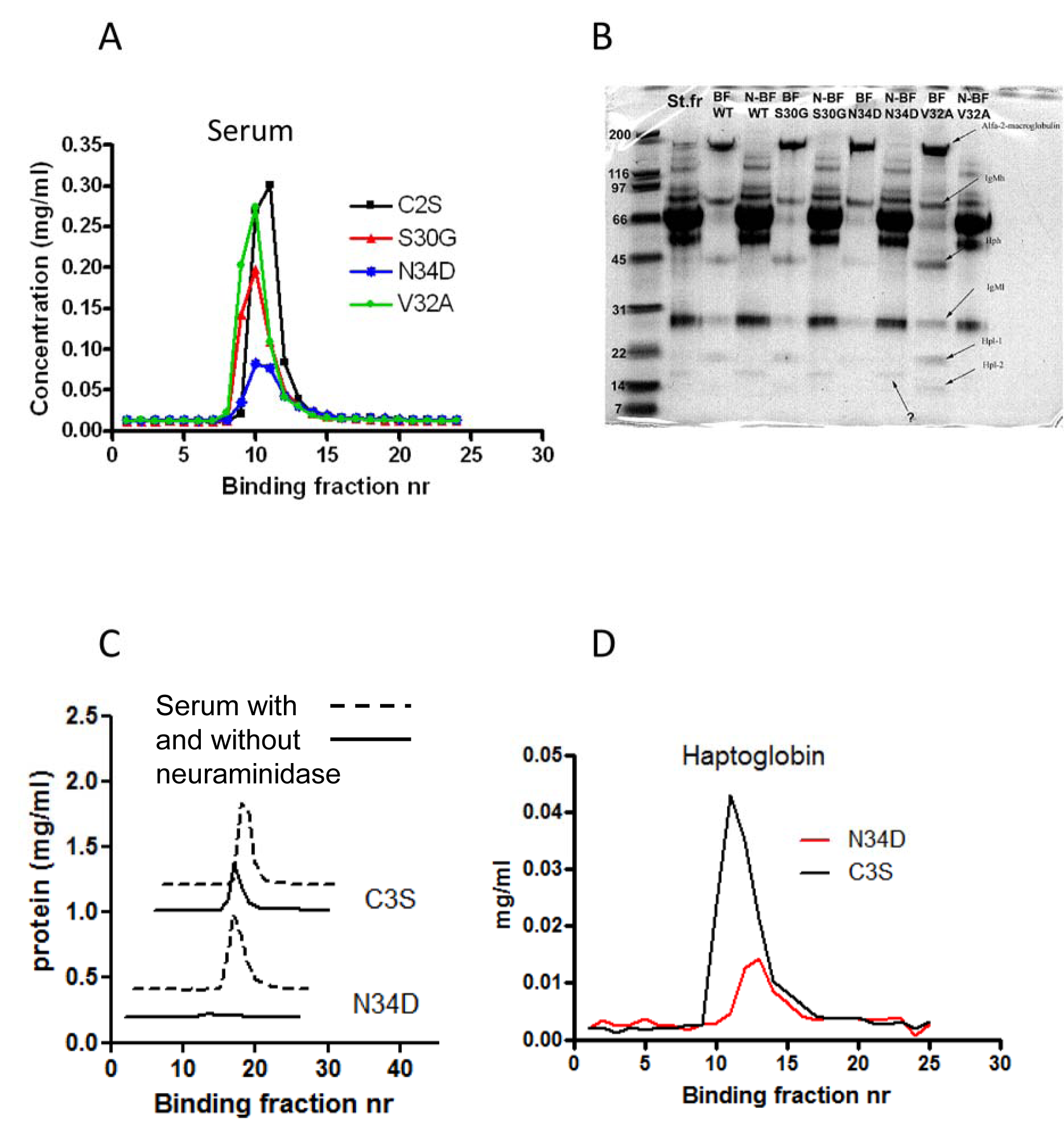

Supplement: Figure S5 — Affinity chromatography of serum and haptoglobin on galectin-1 C3S mutants. (A) Serum from a healthy individual was analyzed using galectin-1 C3S and three further mutants in site B. Only the lactose eluted fractions are shown. Conditions are identical as in Fig. 2. (B). SDS-PAGE of peak bound (BF) and unbound (N-BF) fractions from the experiment of (A). The unfractionated serum (St.fr.) and size markers are shown to the left. All bound fractions showed the same pattern of proteins with α-2-macroglobulin, IgM and haptoglobin as major species, except that mutant N34D bound less of all, most clearly visible for haptoglobin. (C) Affinity chromatography on galectin-1 C3S and C3S N34D of serum treated or not treated with Vibrio cholerae neuraminidase. N34D binds much less untreated serum glycoproteins, but neuraminidase restores binding to equal levels as for galectin-1 C3S. (D) Affinity chromatography of haptoglobin (from pooled human plasma (Sigma-Aldrich)) on galectin-1 C3S and C3S N34D. (TIF) [file pone.0026560.s005.tif]

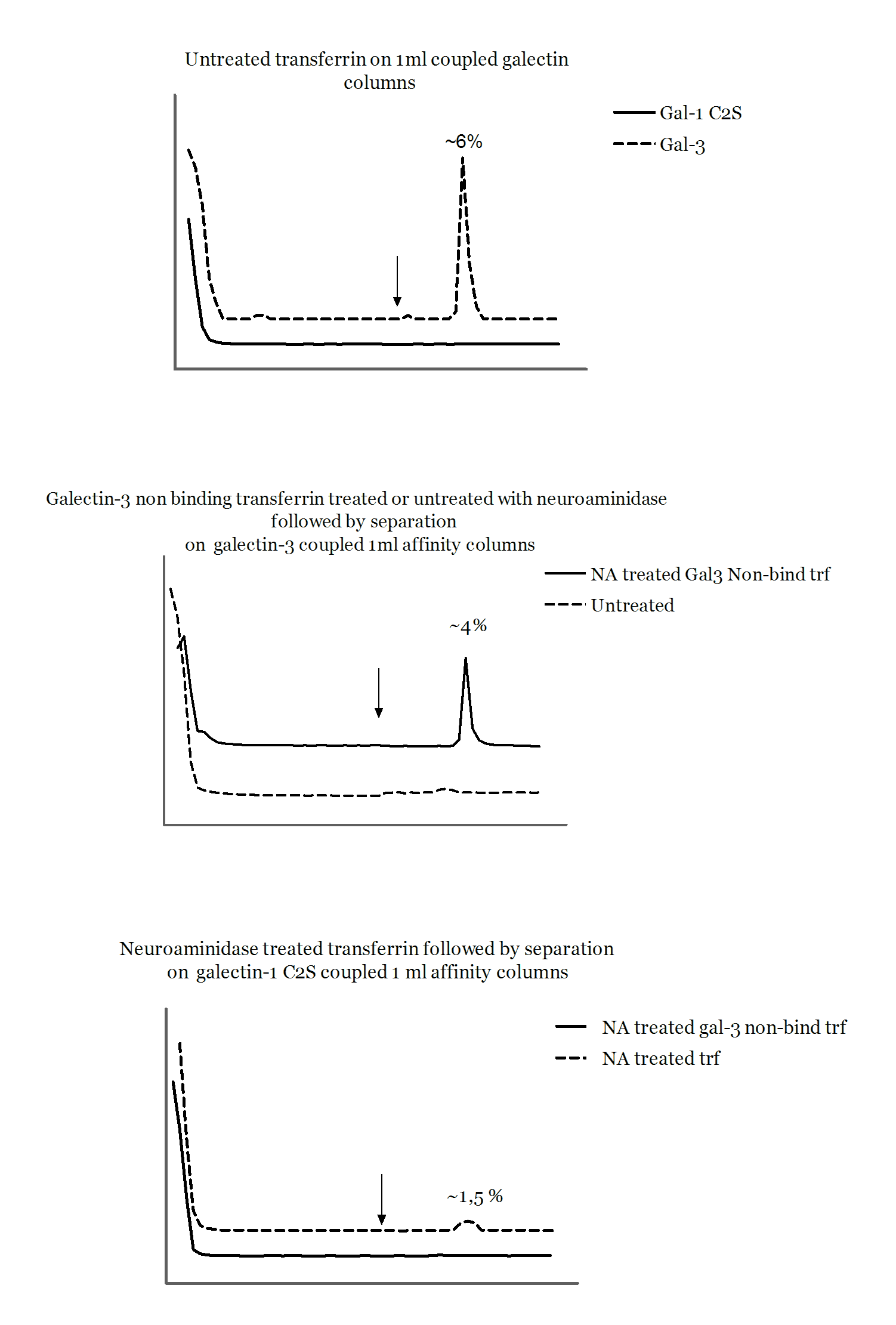

Supplement: Figure S6 — Human transferrin analyzed by affinity chromatography on galectin-1 C3S or galectin-3. 2 mg of human transferrin were analyzed using galectin-1 and galectin-3 coupled 1 ml affinity columns. As predicted galectin-1 does not bind human transferrin, while approximately 6% bind galectin-3 (top panel). The galectin-3 non-binding transferrin was treated or not treated with Vibrio cholerae neuraminidase (NA) (0.1 µmol 1 h at 37°C), and again analyzed on galectin-3 or galectin-1 coupled affinity columns. Removal of sialylations generated an additional 4% of galectin-3 binding transferrin (middle panel), but only traces (about 1.5%) of galectin-1 binding transferrin (bottom panel). (TIF) [file pone.0026560.s006.tif]

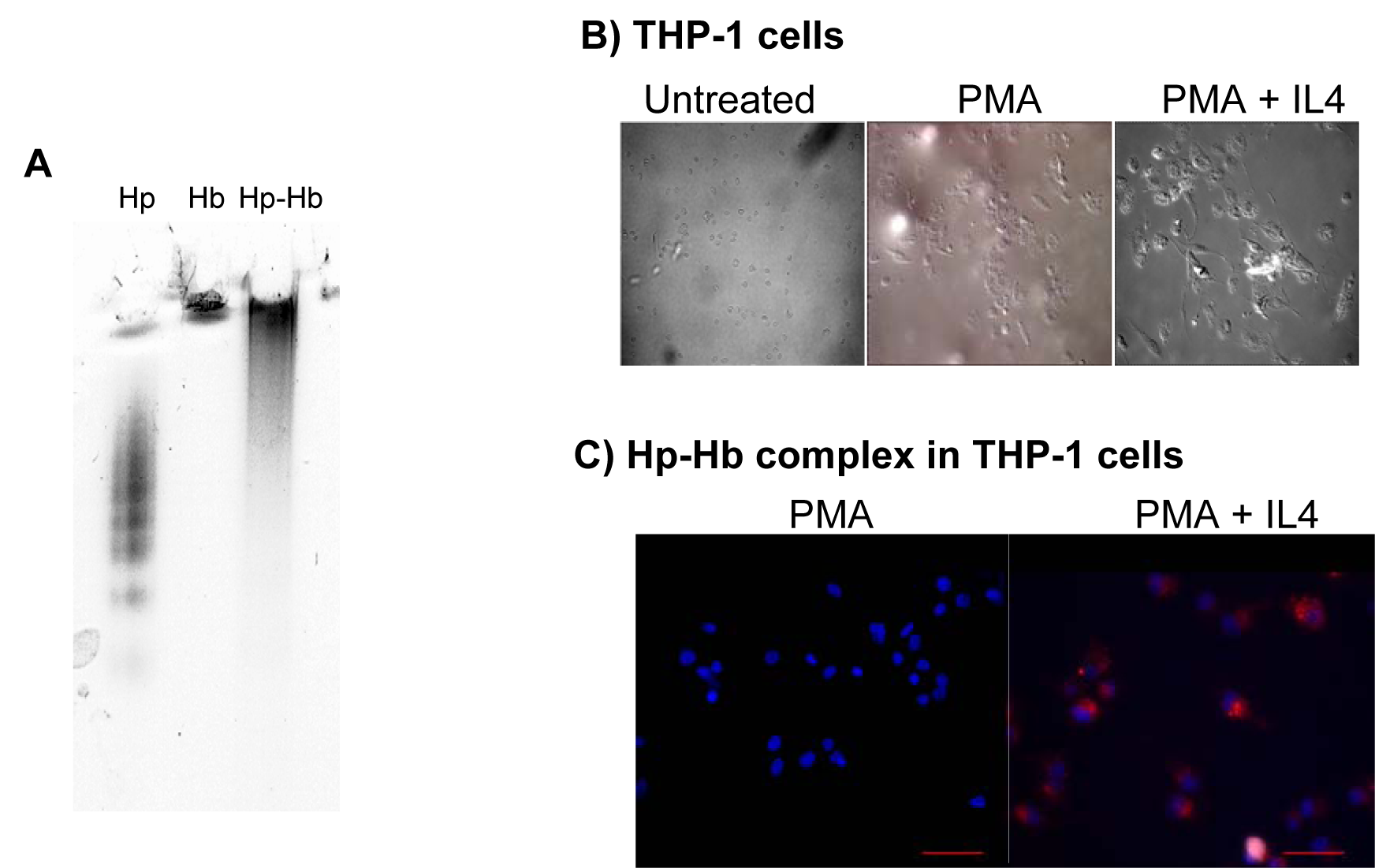

Supplement: Figure S7 — Analysis of haptoglobin-haemoglobin complex in macrophages. A) Native gel electrophoresis of haptoglobin-haemoglobin complex. Haptoglobin (left) moved into the gel, while haemoglobin (middle) did not. For the haptoglobin-haemoglobin complex (right) the free haptoglobin band had disappeared from the gel, indicating that the complex had formed. B) Light microscope images (40×) of THP-1 cells, untreated, induced to differentiate with PMA (5 days), or in addition IL-4 (2 days) for alternatively activation. Untreated cells were transferred to a polylysine glass slide and allowed to sediment for 15 min at room temperature before microscopy, while treated cells were grown directly on coverslips. Scale bar represents 50 µm. C) Uptake of Hp-Hb complex in differentiated and activated THP-1 cells. THP-1 grown in the presence of PMA (5 days) or PMA (5 days) +IL-4 (2 days) were incubated with 0.2 µm NHS-sulphorhodamine conjugated galectin-1 non-binding haptoglobin in complex with haemoglobin for 30 minutes. Cells were fixed in formaldehyde and analyzed by fluorescence microscopy (40×). Nuclei were stained blue with Hoechst. Scale bar represents 50 µm. All microscopy images were taken with a Nikon Eclipse TE2000-E fluorescence microscope. (TIF) [file pone.0026560.s007.tif]

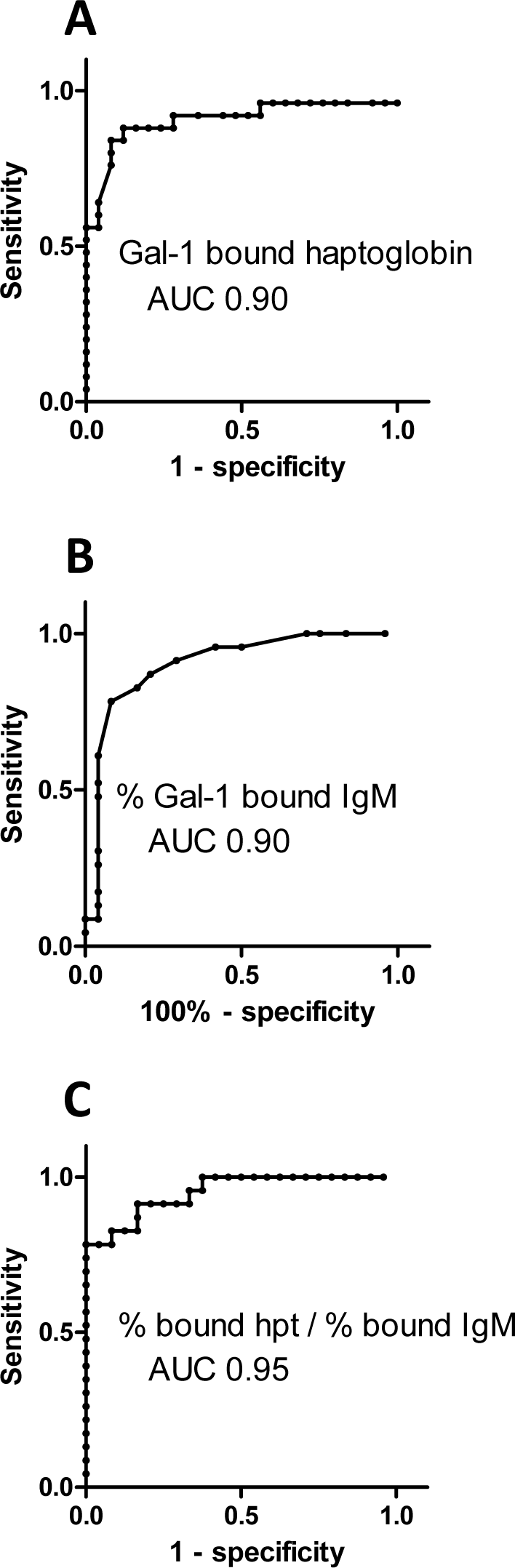

Supplement: Figure S8 — Receiver operating characteristic (ROC) curve analysis for different measured parameters to distinguish sera from breast cancer patients from controls. A) Concentration of galectin-1 bound haptoglobin, B) percentage of galectin-1 bound IgM, and C) ratio of the percentages of galectin-1 bound haptoglobin and IgM. The area under the curve (AUC) indicates the discriminatory power of the measured parameter. A value >0.90 is considered excellent. (TIF) [file pone.0026560.s008.tif]

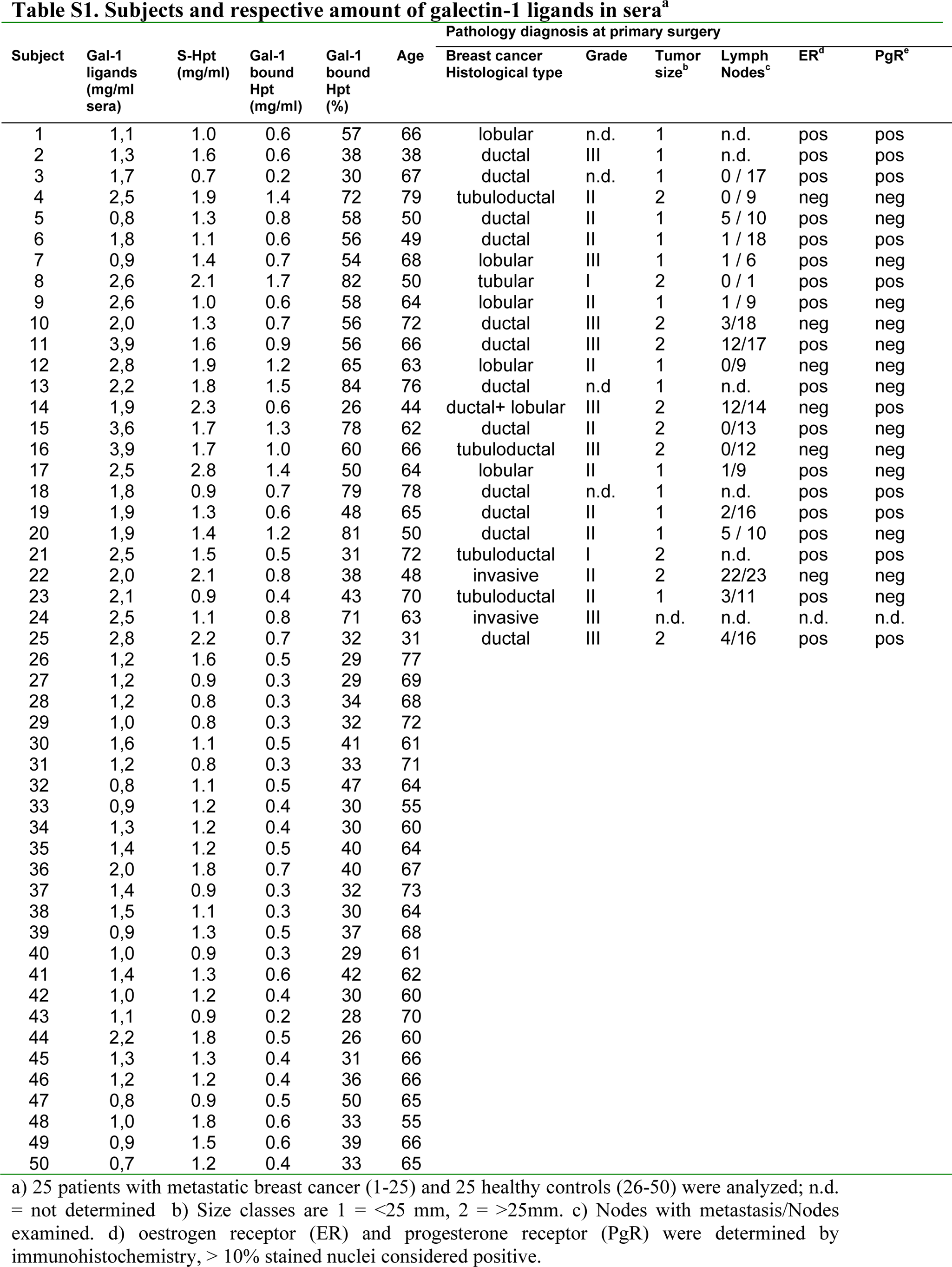

Supplement: Table S1 — Subjects and respective amount of galectin-1 ligands in sera. (TIF) [file pone.0026560.s009.tif]
