# Supplementary material for: Galectin-1-Binding Glycoforms of Haptoglobin with Altered Intracellular Trafficking, and Increase in Metastatic Breast Cancer Patients
Source: PLoS One. 2011 Oct 18;6(10):e26560. doi: 10.1371/journal.pone.0026560 (PMC3196588; doi:10.1371/journal.pone.0026560)

A

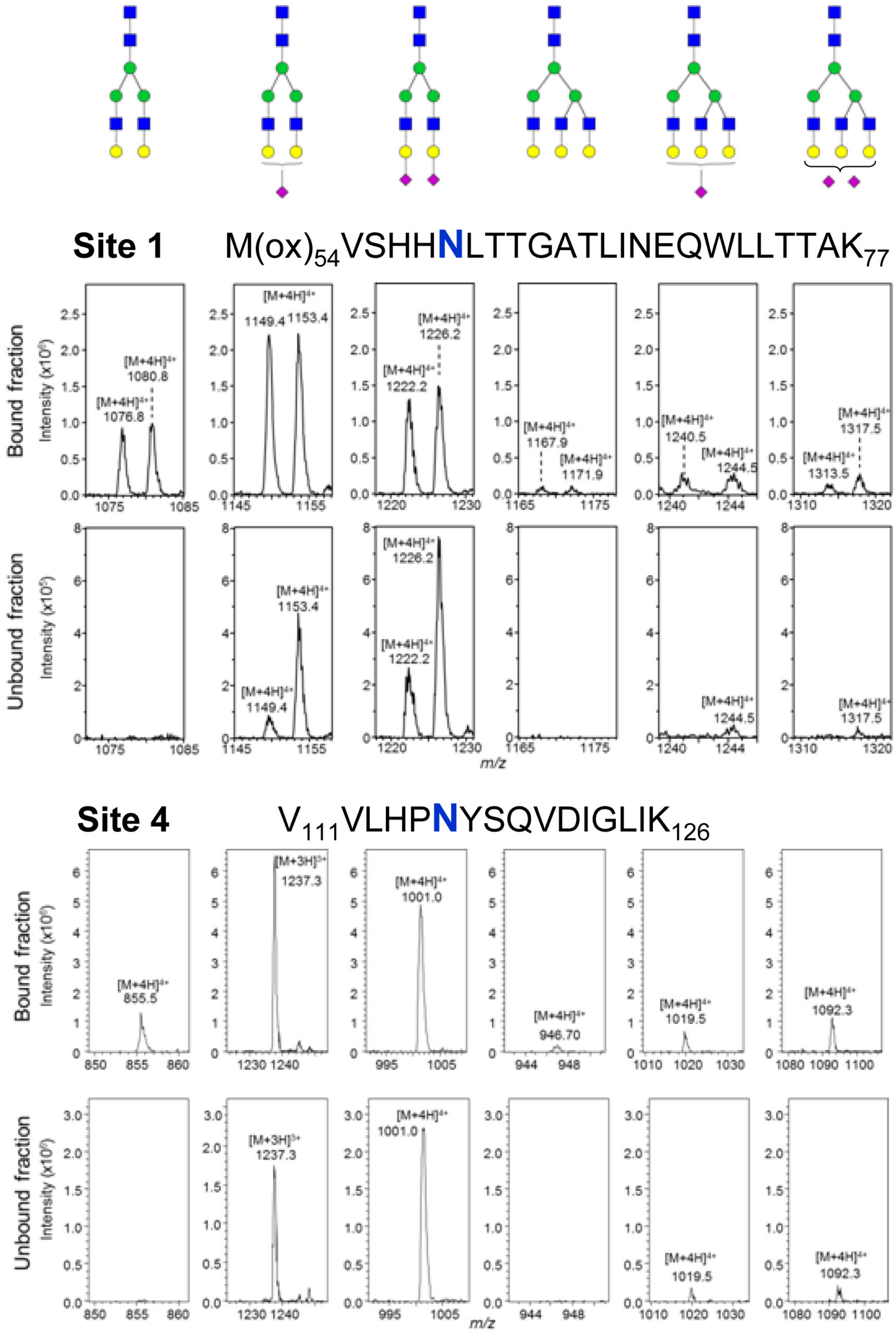

Site 1
M(ox)54VSHHNLTTGATLINEQWLLTTAK77
Bound fraction
Intensity (x10⁵)
[M+4H]4+ 1076.8
[M+4H]4+ 1080.8
[M+4H]4+ 1149.4 1153.4
[M+4H]4+ 1222.2
[M+4H]4+ 1226.2
[M+4H]4+ 1167.9
[M+4H]4+ 1171.9
[M+4H]4+ 1240.5
[M+4H]4+ 1244.5
[M+4H]4+ 1313.5
[M+4H]4+ 1317.5
Unbound fraction
Intensity (x10⁵)
[M+4H]4+ 1149.4
[M+4H]4+ 1153.4
[M+4H]4+ 1222.2
[M+4H]4+ 1226.2
[M+4H]4+ 1244.5
[M+4H]4+ 1317.5
m/z
Site 4
V111VLHPNYSQVDIGLIK126
Bound fraction
Intensity (x10⁵)
[M+4H]4+ 855.5
[M+3H]3+ 1237.3
[M+4H]4+ 1001.0
[M+4H]4+ 946.70
[M+4H]4+ 1019.5
[M+4H]4+ 1092.3
Unbound fraction
Intensity (x10⁵)
[M+3H]3+ 1237.3
[M+4H]4+ 1001.0
[M+4H]4+ 1019.5
[M+4H]4+ 1092.3
m/z

B

Site 2 and 3 $N_{78}LFL\mathbf{N}HSE\mathbf{N}ATAK_{90}$

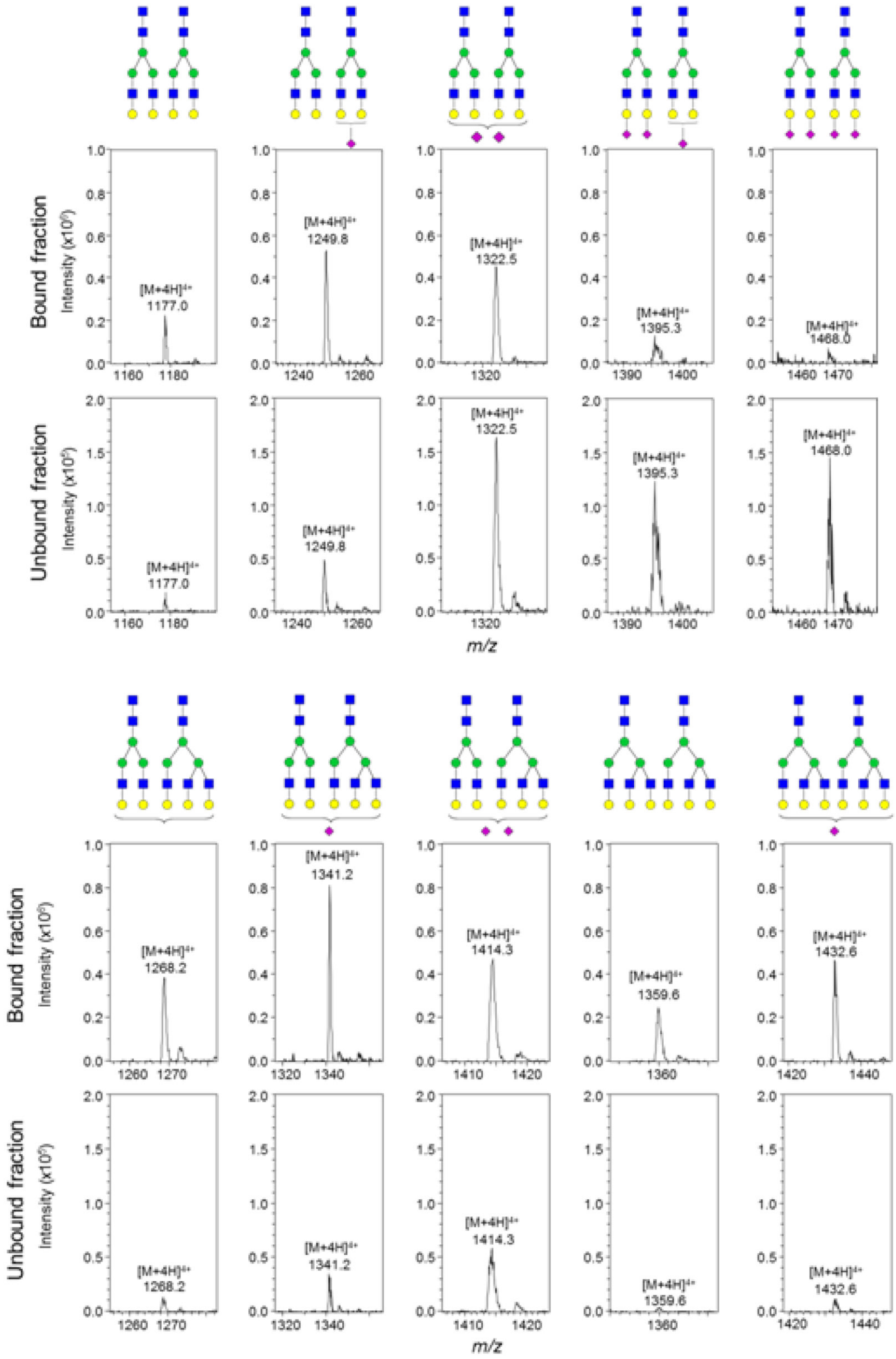

Supplement: Figure S3 — A. Glycosylation analysis of site 1 (N59) or 4 (N116) of galectin-1 C3S bound and unbound haptoglobin. Tryptic glycopeptides were identified by nano-LC-ESI-ion trap-MS, and MS peaks are shown for those carrying the six major N-glycans (schematics above with symbols as in Fig. 5). For site 1 (N59) they were detected as double peaks due to partial oxidization (ox) of methionine giving a 16 Da mass shift. B. Glycosylation analysis of site 2 (N59) and 3 (N116) of galectin-1 C3S bound and unbound haptoglobin. Tryptic glycopeptides containing the two sites were identified by nano-LC-ESI-ion trap-MS, and MS peaks are shown for those carrying the major detected combinations of N-glycans (schematics above mass spectra). (PDF) [file pone.0026560.s003.pdf]
