# Supplementary material for: Galectin-1-Binding Glycoforms of Haptoglobin with Altered Intracellular Trafficking, and Increase in Metastatic Breast Cancer Patients
Source: PLoS One. 2011 Oct 18;6(10):e26560. doi: 10.1371/journal.pone.0026560 (PMC3196588; doi:10.1371/journal.pone.0026560)
Supplement: Table S2 — Relative affinity of galectin-1 proteins for small saccharides and haptoglobin. (DOCX) [file pone.0026560.s010.docx]

| **Inhibitor/Galectin-1** | **C3S** | **N34D** | **V32A** | **S30G** | **R74S** |
| --- | --- | --- | --- | --- | --- |
| **LacNAc^a^** | 1 | 1 | 2 | 0.8 | 1 |
| **NeuAcα2-3LacNAc** | 2 | 0.2 | 1 | 0.4 | nt |
| **GlcNAcβ1-3Lac** | - | -- | 0.5 | -- | nt |
|  |  |  |  |  |  |
| **Haptoglobin^b^** | 2.5 μM | nt | nt | nt | > 20 μM |

**Table S2. Relative affinity of galectin-1 proteins for small saccharides and haptoglobin**

1. Average relative affinities of small saccharides, based on direct binding of fluorescein tagged probes. Binding of galectin-3 to LacNAc-probe (K_d_ ~40 μM) is set as 1. – means not detected and nt not tested
2. Affinity for total haptoglobin containing galectin-1 bound (30%) and unbound part (70%)., based on inhibition of galectin interaction with tdga-probe. The calculation was done using a molecular weight of 120 kD as for dimeric haptoglobin. From this it can be estimated that the affinity for galectin-1 bound haptoglobin is K_d_ < 1 μM, and even lower if higher oligomers are considered.
